# Optimally Biomimetic Passivity-Based Control of a Lower-Limb Exoskeleton Over the Primary Activities of Daily Life

**Published:** 2022-04-12

**Authors:** JIANPING LIN, NIKHIL V. DIVEKAR, GRAY C. THOMAS, ROBERT D. GREGG

**Affiliations:** 1 Robotics Institute, University of Michigan, Ann Arbor, MI 48109 USA; 2 Department of Electrical Engineering and Computer Science, University of Michigan, Ann Arbor, MI 48109 USA

**Keywords:** Biomedical, optimization, robotics

## Abstract

Task-specific, trajectory-based control methods commonly used in exoskeletons may be appropriate for individuals with paraplegia, but they overly constrain the volitional motion of individuals with remnant voluntary ability (representing a far larger population). Human-exoskeleton systems can be represented in the form of the Euler-Lagrange equations or, equivalently, the port-controlled Hamiltonian equations to design control laws that provide *task-invariant* assistance across a continuum of activities/environments by altering energetic properties of the human body. We previously introduced a port-controlled Hamiltonian framework that parameterizes the control law through basis functions related to gravitational and gyroscopic terms, which are optimized to fit normalized able-bodied joint torques across multiple walking gaits on different ground inclines. However, this approach did not have the flexibility to reproduce joint torques for a broader set of activities, including stair climbing and stand-to-sit, due to strict assumptions related to input-output passivity, which ensures the human remains in control of energy growth in the closed-loop dynamics. To provide biomimetic assistance across all primary activities of daily life, this paper generalizes this energy shaping framework by incorporating vertical ground reaction forces and global planar orientation into the basis set, while preserving passivity between the human joint torques and human joint velocities. We present an experimental implementation on a powered knee-ankle exoskeleton used by three able-bodied human subjects during walking on various inclines, ramp ascent/descent, and stand-to-sit, demonstrating the versatility of this control approach and its effect on muscular effort.

## I. INTRODUCTION

State-of-the-art powered exoskeletons are mainly controlled by tracking pre-defined reference trajectories, such as ReWalk [1], Ekso Bionics [2], and Wandercraft [3]. Despite their promising results in gait rehabilitation, significant challenges remain in the control design. The state-of-the-art exoskeletons mentioned above provide complete assistance with trajectory-based, kinematic control methods appropriate for paraplegia. These kinematic control methods replicate the normative joint kinematics associated with one specific task and user at a time [4]. However, the control structures enforce trajectories defined in a database, which cannot adjust to continuously varying tasks and volitional motion of people with remnant voluntary ability, e.g., due to advanced age, stroke, multiple sclerosis, etc. Moreover, these devices have to detect human locomotor intent accurately to transition from one task-dependent controller to another [1], [5], which is hard to realize in practice. The associated parameter tuning for multiple controllers requires more time for each subject and task, and re-tuning becomes necessary as the user progresses through gait therapy.

Fortunately, *backdrivable* exoskeletons [6]–[12] are now enabling a paradigm shift from task-specific, kinematic control approaches to task-invariant, torque control approaches that deliver *partial* rather than *complete* assistance to the user. Various assistive controllers have been proposed to amplify or augment voluntary human motion [12]–[16] or compensate for exoskeleton mass/inertia [17], [18]. However, the torque controllers in [12], [14], [17] require acceleration feedback or load cells to measure human-robot interaction, which are susceptible to noise and can destabilize the system if there is compliance or backlash in the actuation path. The controller in [18] also focuses on reducing the joint-level gravitational torques instead of considering the whole lower-limb model. On the other hand, energy shaping methods [19]–[22] have the potential to provide task-invariant assistance by altering the dynamic characteristics of the human body, as recently demonstrated in a backdrivable knee-ankle exoskeleton [10]. The dynamics of the body are represented by the Euler-Lagrange equations or, equivalently, the Hamiltonian equations, by which a control law is derived to achieve desired dynamics in closed-loop. Underactuated systems can only achieve closed-loop dynamics that satisfy a set of nonlinear partial differential equations called the *matching conditions*, which determine the achievable form of the closed-loop system’s energy and the existence of a feedback law that matches the original control system to the desired closed-loop system. Our prior work on potential energy shaping based on the controlled Lagrangian method provided virtual body-weight support (BWS) during walking in [23], [24]. To compensate for the inertia of the human limbs, we considered total energy shaping (TES) in [25], [26], where kinetic energy was modified through the mass/inertia matrices in addition to the modified potential energy. However, these methods had challenges with ensuring the existence of well-defined, closed-loop kinetic and potential energies in the presence of underactuation. These energy quantities are necessary to preserve passivity between the human muscular inputs and the human joint velocity outputs, which guarantees the change of the system energy is bounded by the energy injected through the input [27]. Passivity implies the human controls the energy growth of the coupled human-exoskeleton system and enables proofs of stability under assumptions of human impedance control [26]. However, underactuation prevents all parts of the mass/inertia matrix from being modified, risking a matrix singularity that prevents a well-defined kinetic energy and thus violates passivity [25]. Underactuation similarly prevents modification of all parts of the gravitational torques vector, possibly preventing the existence of a well-defined potential energy in closed loop. We later demonstrated that a closed-loop potential energy can be achieved by simply adding virtual springs, and velocity-dependent damping terms can be injected without modifying the inertia matrix (i.e., indirect kinetic energy shaping) [28]. Despite the promising simulation results, the indirect kinetic terms were limited by the range of the virtual spring stiffness in practice [28], so significant improvements could not be achieved over the potential energy shaping method.

Our recent work in [29] derived an energy-shaping exoskeleton control strategy based on the Interconnection and Damping Assignment Passivity-based Control (IDA-PBC) method [21], [22], which exploits the interconnection structure of the port-controlled Hamiltonian equations. This method enabled additional velocity-dependent modifications to the dynamics without changing the mass/inertia matrix. The control law depended on basis functions corresponding to gravitational and gyroscopic forces. Providing a fraction of the normative joint torque offloads musculature as in [7], [9], but in a time-invariant, state-based manner. Our prior work [29] optimized the basis functions to fit weight-normalized able-bodied joint torques across walking gaits on different ground inclines. However, this approach was not flexible enough to reproduce joint torques for a broader set of activities, including stair climbing and stand-to-sit. Modifications to the gravitational torques vector in [29] depended only on the actuated coordinates, as a convenient way to prove the existence of a closed-loop potential energy and thus passivity and stability. Without additional feedback like the leg’s orientation or ground reaction forces (GRFs), the controller was limited to nonlinear spring-like behavior.

This paper generalizes our prior IDA-PBC method to include unactuated coordinates such as leg orientation in a passivity-based, energy-shaping controller for optimal assistance of all primary activities of daily life (ADLs). In addition to global orientation, we include the vertical GRF in the basis functions to address prior problems with excessive torque as weight transfers from the assisted leg to the (unmodeled) contralateral leg during double support [24]. Incorporating these additional variables increases the candidate basis functions in the optimization process, enabling the controller to fit normalized able-bodied human joint torques more closely across more activities, including stand-to-sit and stair climbing tasks. This optimization process leverages “L1 regularization” to fit the data with as few parameters as possible to avoid overfitting with the additional basis functions. We formulate and solve this optimization problem using convex programming tools. The resulting controller is assessed in terms of the similarity to normalized able-bodied human torques in a data-driven simulation. This simulation returns the optimal parameters for the controller to provide proper torque assistance for multiple tasks. We apply these parameters to a real-time implementation of the controller on a powered knee-ankle exoskeleton used by multiple human subjects to demonstrate feasibility of the proposed multi-task optimized energy shaping method.

The contributions of this paper are summarized as follows. First, we generalize our optimization-based energy-shaping control framework based on the port-controlled Hamiltonian equations by incorporating global planar orientation and GRFs in the basis functions, while preserving input-output passivity and stability for safe human-robot interaction. Second, this framework enables a single feedback controller to closely fit normalized able-bodied human joint torques for all primary ADLs: level-ground walking, walking at variable inclines/declines, stair ascent/descent with variable step heights, and stand-to-sit. While recent work in [30] proposed a deep learning approach to estimating subject-independent hip joint torques for walking on level ground, ramps, and stairs, no prior work has estimated more complicated knee and ankle torque profiles for all primary ADLs (without switching or adaptation between tasks). Third, we assess the muscular effort of multiple able-bodied human subjects with an experimental implementation of this task-invariant control method on a (knee-ankle) exoskeleton to assist the primary ADLs.

The rest of this paper is organized as follows. Section II reviews the concepts of the port-controlled Hamiltonian systems and the corresponding matching conditions for the human-exoskeleton dynamics with contact constraints. In Section III, we design the desired closed-loop Hamiltonian system and the corresponding control law by incorporating the global orientation variable and GRF. We highlight passivity and stability properties based on common human control policies. Section IV presents training and validation results for the optimized controller over a dataset of the primary ADLs. Section V then presents the hardware implementation and able-bodied human subject experiments. Finally, we summarize the limitation of the proposed study and provide possible future research directions.

## II. REVIEW OF ENERGY SHAPING CONTROL FOR EXOSKELETONS

This section briefly reviews interconnection and damping assignment passivity-based control (IDA-PBC) for the human-exoskeleton system in [29]. We present the solution to the matching conditions with contact constraints, define the corresponding control law, and define input-output passivity.

### A. PORT-CONTROLLED HAMILTONIAN DYNAMICS

**FIGURE 1.**
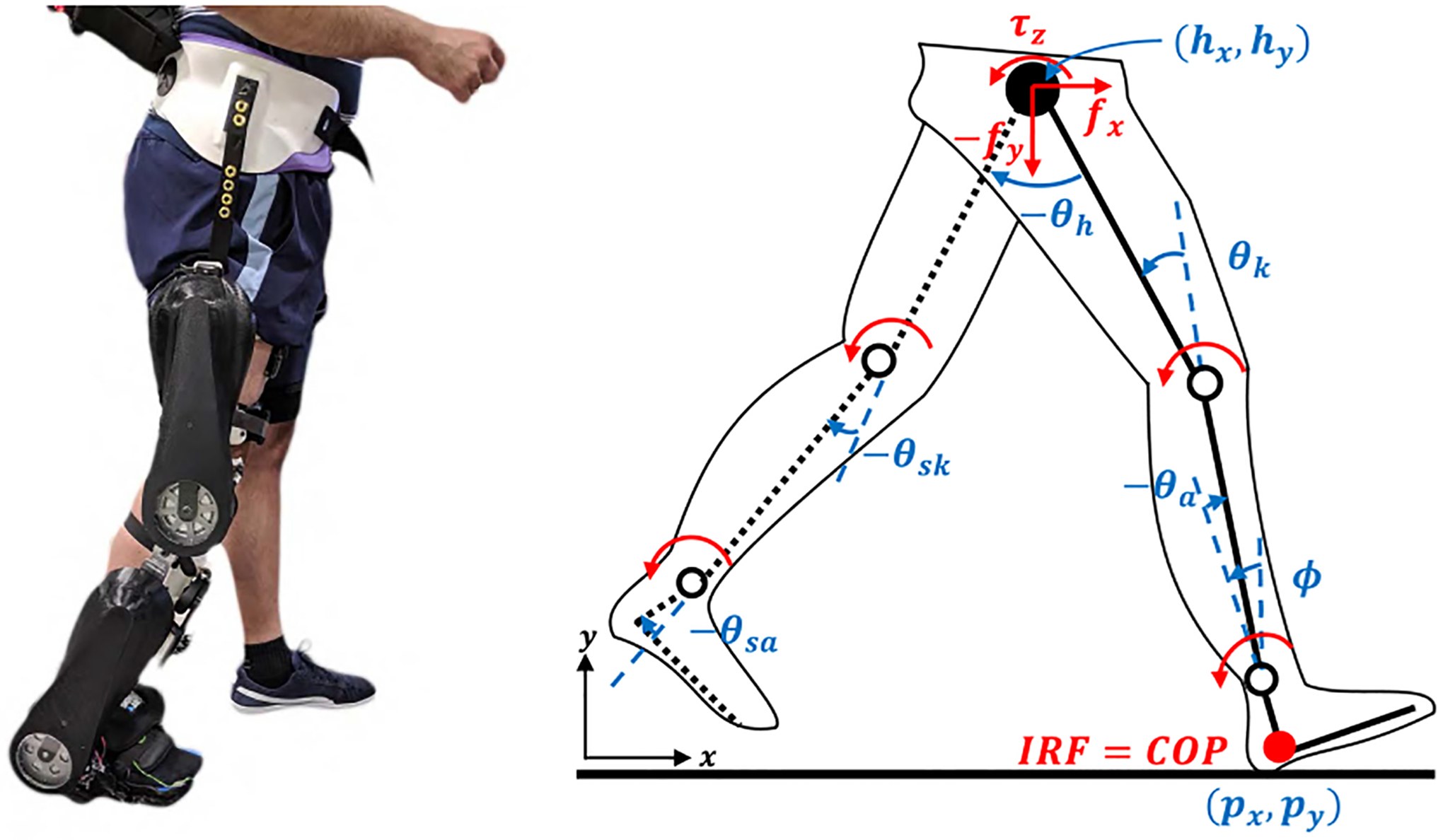
Left: *Comex* knee-ankle exoskeleton worn by a healthy user (reproduced from [24]). Right: Kinematic model of the human body (reproduced from [28]). COP denotes Center of Pressure. Solid links denote the stance leg, and dashed links denote the swing leg. Red arcs indicate torques.

We consider a 6-link sagittal plane human-exoskeleton biped model with a floating stance foot and five revolute joints (Fig. 1). The inertial reference frame (IRF) is coincident with the position of the heel, (*p*_*x*_, *p*_*y*_), during the heel contact phase. The global heel angle *ϕ* is defined with respect to the vertical axis. The stance ankle and knee angles are denoted by *θ*_*a*_ and *θ*_*k*_, respectively. The inter-leg angle between the stance thigh and the swing thigh is denoted by *θ*_*h*_, and the swing knee and ankle angles are *θ*_*sk*_ and *θ*_*sa*_, respectively. The masses and moments of inertia in the model reflect the combination of the human and exoskeleton masses.

For the purpose of control derivation, the dynamics of the stance and swing legs are modeled separately with coupled interaction forces $F={\left[f_{x},f_{y},\tau_{z}\right]}^{T}\in {\mathbb{R}}^{3\times 1}$. The five degree-of-freedom (DOF) stance leg model has the generalized coordinates $q={\left[p_{x},p_{y},\phi ,\theta_{a},\theta_{k}\right]}^{T}\in {\mathbb{R}}^{5\times 1}$ in the 5-dimensional configuration space Q (solid in Fig. 1). The conjugate momenta $p=M(q)\dot{q}\in {\mathbb{R}}^{5\times 1}$ are defined by the positive-definite inertia matrix $M(q)\in {\mathbb{R}}^{5\times 5}$ and the velocity vector $\dot{q}$. The port-controlled Hamiltonian dynamics can be characterized by the Hamiltonian $H(q,p):T^{*}Q\to \mathbb{R}$ through the equations

(1) $$\left[\begin{array}{c} \dot{q}\\ \dot{p} \end{array}\right]=\left[\begin{array}{cc} 0_{5\times 5} & I_{5\times 5}\\ -I_{5\times 5} & 0_{5\times 5} \end{array}\right]\nabla H+\left[\begin{array}{c} 0_{5\times 1}\\ \tau +A^{T}\lambda \end{array}\right],$$

where the skew-symmetric matrix above is known as the interconnection matrix. The Hamiltonian function $H(q,p)=\frac{1}{2}p^{T}M^{-1}(q)p+V(q)$ is given by the kinetic plus potential energy V(q)∈ℝ. The gradient ∇*H* = [*∂*_*q*_*H*, *∂*_*p*_*H*]^*T*^ is a column vector in ${\mathbb{R}}^{10\times 1}$ with *∂*_*q*_*H*, $\partial_{p}H\in {\mathbb{R}}^{1\times 5}$ as row vectors. The vector of joint torques $\tau \in {\mathbb{R}}^{5\times 1}$ aggregates the exoskeleton input *τ*_exo_ = *Gu* and the human input *τ*_hum_ = *Gυ* + *J*(*q*)^*T*^ *F*. The control inputs $u\in {\mathbb{R}}^{2\times 1}$ and $v\in {\mathbb{R}}^{2\times 1}$ respectively represent the exoskeleton and human torques (at the knee and ankle joints), which are mapped into the overall dynamics via matrix $G\in {\mathbb{R}}^{5\times 2}$. The system is underactuated with the number of generalized coordinates larger than the number of control inputs. The interaction forces *F* are mapped into the system’s dynamics by the Jacobian matrix $J(q)\in {\mathbb{R}}^{3\times 5}$.

**FIGURE 2.**
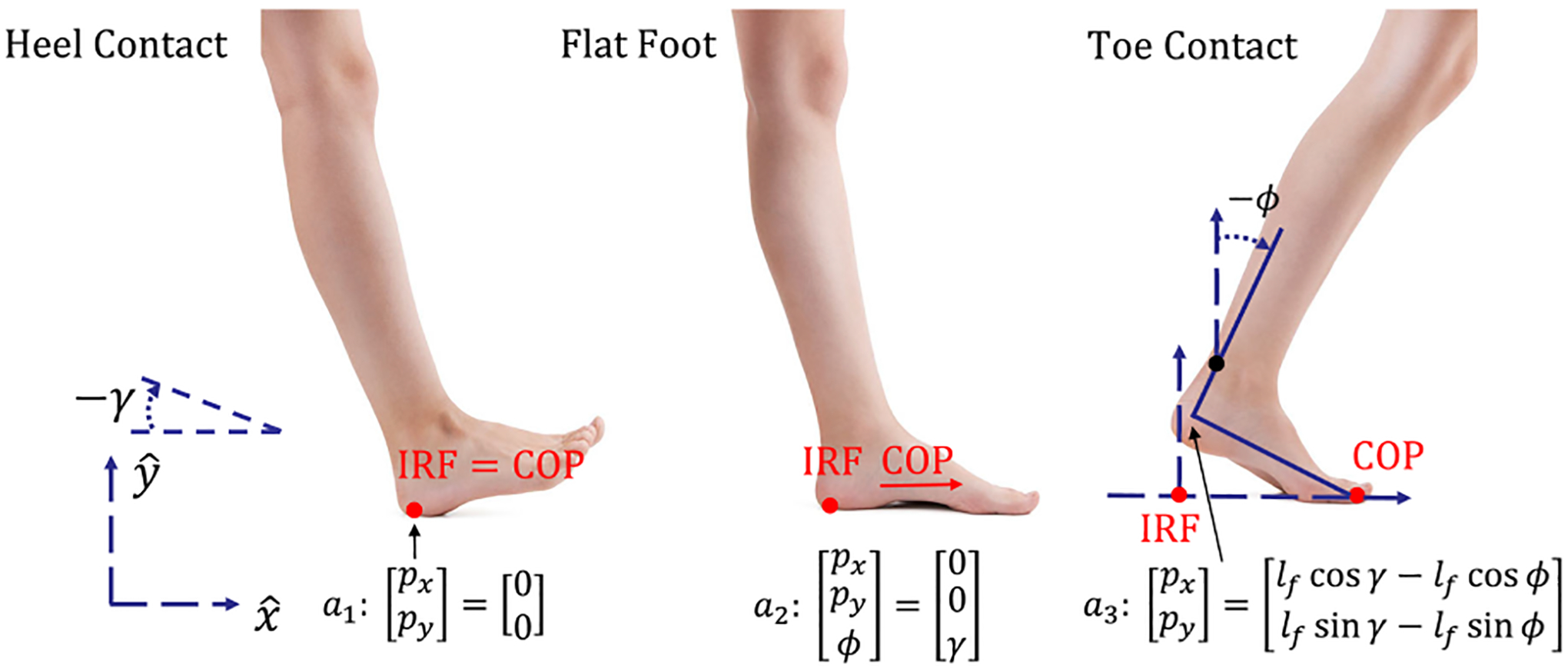
Heel contact (left), flat foot (center), and toe contact (right) during the single-support period of human locomotion. Angle *γ* is the ground slope. This figure is updated from [10].

The holonomic contact constraints in the human-exoskeleton dynamics (Fig. 2) can be expressed as *a*_*ℓ*_(*q*) = 0_*c*×1_, where *c* is the number of constraints and the subscript *ℓ* ∈ {heel, flat, toe} indicates the contact configuration. The constraint matrix $A(q)=\partial_{q}a_{\ell }\in {\mathbb{R}}^{c\times 5}=\left[A_{\ell }\;0_{c\times 2}\right]$ satisfies $A\dot{q}=A{\left(\partial_{p}H\right)}^{T}=0$ given the top row of (1). The possible cases are

$$\text{Heel Contact }A_{\text{heel}}(q)=\left[\begin{array}{ll} I_{2\times 2} & 0_{2\times 1} \end{array}\right],$$

$$\text{Flat Foot }A_{\text{flat}}(q)=I_{3\times 3},\text{ and}$$

$$\text{Toe Contact }A_{\text{toe}}(q)=\left[\begin{array}{ccc} 1 & 0 & -l_{f}\text{ sin}(\phi )\\ 0 & 1 & l_{f}\text{ cos}(\phi ) \end{array}\right],$$

where *γ* is the slope angle and *l*_*f*_ is the length of the foot. The Lagrange multiplier $\lambda \in {\mathbb{R}}^{c\times 1}$ represents the GRFs, which are mapped into the system through the constraint matrix *A*. Details for the contact constraints are given in [10], [23]. Henceforth we omit *q* and *p* terms in matrices to simplify notation.

The Lagrange multiplier λ can then be obtained by solving $\frac{d}{dt}\left(A{\left(\partial_{p}H\right)}^{T}\right)=0\to \partial_{q}\left[A{\left(\partial_{p}H\right)}^{T}\right]\dot{q}+\partial_{p}\left[A{\left(\partial_{p}H\right)}^{T}\right]\dot{p}=0$ for

$$\lambda ={\left(A\partial_{p^{2}}^{2}HA^{T}\right)}^{-1}\left\{-\partial_{q}\left[A{\left(\partial_{p}H\right)}^{T}\right]{\left(\partial_{p}H\right)}^{T}+A\partial_{p^{2}}^{2}H\left[{\left(\partial_{q}H\right)}^{T}-\tau \right]\right\},$$

where $\partial_{p^{2}}^{2}H\in {\mathbb{R}}^{5\times 5}$ denotes the second-order derivative of *H* with respect to *p*.

For the swing leg model (dotted in Fig. 1), the configuration is given by *q*_*s*w_ = [*h*_*x*_, *h*_*y*_, *θ*_*th*_, *θ*_*sk*_, *θ*_*sa*_]^*T*^, where (*h*_*x*_, *h*_*y*_) are the positions of the hip with respect to the IRF. The angle between the vertical axis and the swing thigh is denoted as *θ*_*th*_. The swing leg dynamics do not have contact constraints.

### B. CONTROL LAW SATISFYING THE MATCHING CONDITIONS

Assume we have closed the feedback loop for exoskeleton input *u*, while the human inputs *υ* and *F* remain open-loop in the Hamiltonian system. We consider a desired, closed-loop Hamiltonian $\tilde{H}(p,q)=\frac{1}{2}p^{T}{\tilde{M}}^{-1}p+\tilde{V}$, where $\tilde{V}=V+\hat{V}$ represents the new potential energy with shaping term $\hat{V}$. The corresponding gravitational vector is $\tilde{N}={\left(\partial_{q}\tilde{V}\right)}^{T}={\left(\partial_{q}V\right)}^{T}+{\left(\partial_{q}\hat{V}\right)}^{T}=N+\hat{N}\in {\mathbb{R}}^{5\times 1}$. We set $\tilde{M}=M$ to simplify the matching process and passivity proof, and to avoid complicated calculations of the inertia matrix inverse in the control law. This implies $\nabla \tilde{H}=\nabla H+{\left[\partial_{q}\hat{V},0\right]}^{T}$ but we can still achieve velocity-dependent shaping by modifying the interconnection matrix of the closed-loop Hamiltonian system.

The desired closed-loop dynamics based on $\tilde{H}$ are

(2) $$\left[\begin{array}{c} \dot{q}\\ \dot{p} \end{array}\right]=\left[\begin{array}{cc} 0 & I\\ -I & J_{2} \end{array}\right]\nabla \tilde{H}+\left[\begin{array}{c} 0\\ Gv+J^{T}F+A^{T}\tilde{\lambda }+T_{\text{ex}} \end{array}\right],$$

where $T_{\text{ex}}\in {\mathbb{R}}^{5\times 1}$ denotes the new exogenous input compared to [29]. The skew-symmetric matrix $J_{2}=-J_{2}^{T}\in {\mathbb{R}}^{5\times 5}$ represents the extra shaping DOF provided in the interconnection structure by the IDA-PBC method [29]. This introduces *artificial* gyroscopic terms *Q*^*T*^ (*∂*_*p*_*H*)^*T*^, where $Q(q)\in {\mathbb{R}}^{5\times 1}$ is a smooth vector-valued function and *J*_2_ = (*∂*_*q*_*Q*)^*T*^ − *∂*_*q*_*Q*. Moreover, the closed-loop GRFs in (2) are represented by

$$\tilde{\lambda }={\left(A\partial_{p^{2}}^{2}HA^{T}\right)}^{-1}\left\{-\partial_{q}\left[A{\left(\partial_{p}H\right)}^{T}\right]{\left(\partial_{p}H\right)}^{T}+A\partial_{p^{2}}^{2}H\left[{\left(\partial_{q}\tilde{H}\right)}^{T}-J_{2}{\left(\partial_{p}H\right)}^{T}-Gv-J^{T}F-T_{\text{ex}}\right]\right\}.$$

Based on standard results in [20], Hamiltonian systems (1) and (2) *match* if we have

$$Gu=-{\left(\partial_{q}\tilde{H}\right)}^{T}+{\left(\partial_{q}H\right)}^{T}+J_{2}{\left(\partial_{p}H\right)}^{T}+A^{T}(\tilde{\lambda }-\lambda )+T_{\text{ex}}.$$

By plugging GRFs λ and $\tilde{\lambda }$ and following the steps in [29], we have

(3) $$G_{\lambda }u=X_{\lambda }\left[-{\left(\partial_{q}\tilde{H}\right)}^{T}+{\left(\partial_{q}H\right)}^{T}+J_{2}{\left(\partial_{p}H\right)}^{T}+T_{\text{ex}}\right],$$

and the corresponding *matching condition:*

(4) $$0=G_{\lambda }^{⊥}X_{\lambda }\left[-{\left(\partial_{q}\tilde{H}\right)}^{T}+{\left(\partial_{q}H\right)}^{T}+J_{2}{\left(\partial_{p}H\right)}^{T}+T_{\text{ex}}\right],$$

where $G_{\lambda }^{⊥}\in {\mathbb{R}}^{3\times 5}$ is the (full-rank) left annihilator of *G*_λ_ = *X*_λ_*G*, i.e., $G_{\lambda }^{⊥}G_{\lambda }=0$, and $X_{\lambda }=I-A^{T}WA\partial_{p^{2}}^{2}H\in {\mathbb{R}}^{5\times 5}$ with $W={\left(A\partial_{p^{2}}^{2}HA^{T}\right)}^{-1}\in {\mathbb{R}}^{c\times c}$.

To solve the matching condition (4), we decompose matrix *M* into four sub-matrices as in [29]:

$$M=\left[\begin{array}{ll} M_{1} & M_{2}\\ M_{2}^{T} & M_{4} \end{array}\right],$$

where $M_{1}\in {\mathbb{R}}^{3\times 3}$ corresponds to the floating base joints (*p*_*x*_, *p*_*y*_, *ϕ*) and $M_{4}\in {\mathbb{R}}^{2\times 2}$ corresponds to the actuated joints (*θ*_*a*_, *θ*_*k*_). Then we obtain

$$M^{-1}=\left[\begin{array}{cc} \Delta^{-1} & -\Delta^{-1}M_{2}M_{4}^{-1}\\ -M_{4}^{-1}M_{2}^{T}\Delta^{-1} & M_{4}^{-1}+M_{4}^{-1}M_{2}^{T}\Delta^{-1}M_{2}M_{4}^{-1} \end{array}\right],$$

where $\Delta =M_{1}-M_{2}M_{4}^{-1}M_{2}^{T}\in {\mathbb{R}}^{3\times 3}$. The solution of the matching condition (4) can be simplified as

(5) $$0=\left[\begin{array}{ll} I-Z_{\lambda } & 0_{3\times 2} \end{array}\right]\left[-{\left(\partial_{q}\tilde{H}\right)}^{T}+{\left(\partial_{q}H\right)}^{T}+J_{2}{\left(\partial_{p}H\right)}^{T}+T_{\text{ex}}\right],\;=\left[\begin{array}{ll} I-Z_{\lambda } & 0_{3\times 2} \end{array}\right]\left[-\tilde{N}+N+J_{2}M^{-1}p+T_{\text{ex}}\right],$$

where $Z_{\lambda }=A_{\ell }^{T}WA_{\ell }\Delta^{-1}\in {\mathbb{R}}^{3\times 3}$ and $W={\left(A_{\ell }\Delta^{-1}A_{\ell }^{T}\right)}^{-1}\in {\mathbb{R}}^{c\times c}$. By zeroing the unactuated parts (first three elements) of $-\tilde{N}+N+J_{2}M^{-1}p+T_{\text{ex}}$, the matching condition (4) is satisfied. More details can be found in [29].

The control law for the feasible shaping structure satisfying (3) is

(6) $$Gu=\left[{\left(\partial_{q}H\right)}^{T}-{\left(\partial_{q}\tilde{H}\right)}^{T}+J_{2}M^{-1}p+T_{\text{ex}}\right]\;u=G^{+}\left(-\hat{N}+J_{2}M^{-1}p+T_{\text{ex}}\right),$$

with *G*^+^ = (*G*^*T*^ *G*)^−1^*G*^*T*^ being the left pseudoinverse of *G*. Note that velocity dependence is introduced via the conjugate momenta *p*. In [29], the exogenous input *T*_ex_ = 0. Moreover, the closed-loop system (2) is integrable with a well-defined potential energy if the unactuated parts of $\hat{N}$ and *Q*(*q*) are zero and the actuated parts depend only on actuated state variables [29, Proposition 1]. Integrability guarantees there exists an equivalent Lagrangian (or Hamiltonian) $\tilde{L}(q,\dot{q})=\frac{1}{2}{\dot{q}}^{T}M\dot{q}+{\dot{q}}^{T}Q-\tilde{V}$ to ensure passivity [27]:

*Definition 2.1:* Consider a general mechanical system

(7) $$\dot{x}=f(x,u),\;y=h(x,u),$$

where $x\in {\mathbb{R}}^{n\times 1},u\in {\mathbb{R}}^{p\times 1}$ is the input and $y\in {\mathbb{R}}^{p\times 1}$ is the output. Let $E(x):{\mathbb{R}}^{n\times 1}\to \mathbb{R}$ be a continuously differentiable, positive semi-definite function, then the system (7) is passive from input *u* to output *y* if $\dot{E}(x)=\frac{\partial E}{\partial x}f(x,u)\leq y^{T}u$.

Input-output passivity means that for a continuously differentiable, positive semi-definite function, the time derivative is restricted by the input times the output. In other words, the change in the system energy is bounded by the energy injected through the input *u*. The system absorbs power but does not generate energy on its own. Having well-defined energy provides a useful storage function *E* for passivity analysis. However, it has previously limited the flexibility of the closed-loop dynamics [29], which we address next.

## III. PASSIVITY-BASED OPTIMAL CONTROLLER DESIGN

The modified gravitational vector $\hat{N}$ in [29, Proposition 1] depends only on the actuated variables to ensure the closed-loop system satisfies matching conditions, *i.e*., the corresponding potential energy must have a zero partial derivative with respect to the unactuated coordinates to avoid applying torque at the unactuated joints. However, this restricts the controller to virtual spring behaviors, limiting its flexibility to reproduce normative joint torques over multiple ADLs.

Instead of restricting the potential energy as in [29], we now pursue a strategy of designing an unrestricted potential energy function. This energy function has a non-zero partial with respect to the unactuated coordinates, so we introduce a new exogenous input *T*_ex_ that cancels out the unactuated component of the joint torques. We call this input a “power leak,” as it can add and remove energy through a port comprising the aforementioned unactuated joint torques and the unactuated joint velocities. Thus we can then incorporate the global variable *ϕ* into the actuated part of $\hat{N}$ and *J*_2_ *M*^−1^*p*, where the matching condition (4) is satisfied.

The corresponding modified potential energy for the stance leg model is denoted as $\hat{V}=\hat{V}\left(\phi ,\theta_{a},\theta_{k}\right)$, where the associated conservative force vector $\hat{N}={\left(\partial_{q}\hat{V}\right)}^{T}={\left[0,0,{\hat{N}}_{3}\left(\phi ,\theta_{a},\theta_{k}\right),{\hat{N}}_{4}\left(\phi ,\theta_{a},\theta_{k}\right),{\hat{N}}_{5}\left(\phi ,\theta_{a},\theta_{k}\right)\right]}^{T}$. We define ${\hat{N}}_{\text{act}}={\left[0,0,0,{\hat{N}}_{4}\left(\phi ,\theta_{a},\theta_{k}\right),{\hat{N}}_{5}\left(\phi ,\theta_{a},\theta_{k}\right)\right]}^{T}$ and the exogenous input $T_{\text{ex}}={\left[0,0,{\hat{N}}_{3}\left(\phi ,\theta_{a},\theta_{k}\right),0,0\right]}^{T}$. Vector ${\hat{N}}_{\text{act}}$ comprises only the actuated components in $\hat{N}$, i.e., ${\hat{N}}_{4}$ and ${\hat{N}}_{5}$ correspond to the conservative force vector acting on the ankle and knee joints. The difference $T_{\text{ex}}=\hat{N}-{\hat{N}}_{\text{act}}$ between the desired torque vector $\hat{N}$ and the applied underactuated torques ${\hat{N}}_{\text{act}}$ can be treated as a new “power leak” port that transfers power into and out of our system. We investigate the energetic influence of this power leak in our passivity analysis of the exoskeleton-human system as follows.

*Proposition 3.1:* If $\hat{V}$ is continuously differentiable, then the closed-loop system (2) is passive with two input ports: the human input with effort *τ*_hum_ and flow $\dot{q}$, and the power leak port with effort ${\hat{N}}_{3}$ and flow $\dot{\phi }$.

*Proof:* Consider the storage function $E=\tilde{H}=H+\hat{V}$ and the closed-loop system (2), where $\partial_{p}\tilde{H}=\partial_{p}H$ and ${\left(\partial_{q}\tilde{H}\right)}^{T}={\left(\partial_{q}H\right)}^{T}+\hat{N}$. The time derivative of *E*(*q*, *p*) is

E˙=(∂qH)q˙+(∂qV^)q˙+(∂pH)p˙=(∂qH)(∂pH)T+N^T(∂pH)T+(∂pH)[−(∂qH)T−N^+τhum+J2(∂pH)T+ATλ˜+Tex]=(∂pH)τhum+(∂pH)Tex+(∂pH)J2(∂pH)T+(∂pH)ATλ˜=q˙Tτhum+ϕ˙N^3,

where we use the skew-symmetry property of the interconnection structure *J*_2_ (the quadratic form with a skew-symmetric matrix is zero), and $\left(\partial_{p}H\right)A^{T}\tilde{\lambda }=0$ due to the fact that constraint forces do no work [31]. Thus, energy growth in the system is controlled by the two input ports. ■

In practice, the power leak results in a small contribution relative to the power input from the human, who essentially controls the power growth of the system alone. This provides safe interaction with the exoskeleton, but stability depends on the human control law. Although *ϕ* is unactuated with respect to the muscles on the ipsilateral leg, the interaction forces with the rest of the body can actuate this DOF (especially during double support phase). We assume that the human is modulating joint impedance [10], [32] and compensating the missing gravitational component in $\hat{N}$, where.

(8) $$\tau_{\text{hum}}=-K_{p}e-K_{d}\dot{e}-{\left[0,0,{\hat{N}}_{3}\left(\phi ,\theta_{a},\theta_{k}\right),0,0\right]}^{T}.$$

The constant diagonal matrices *K*_*p*_, *K*_*d*_ are positive semi-definite, and $e=q-\bar{q}$ represents the difference between *q* and the human’s constant set-point vector $\bar{q}$. We assume these impedance parameters remain (piecewise) constant during small movements, as often modeled in human-inspired finite state machine controllers [18], [32]. We can show the stability of the closed-loop system (2) around the equilibrium point (*q*^⋆^, 0), where the forces along the shaped potential energy balance the muscular forces and the GRFs, i.e., $N+\hat{N}-\tau_{\text{hum}}-A^{T}\tilde{\lambda }(q,0)-T_{\text{ex}}=N+{\left(\partial_{q}\hat{V}\right)}^{T}+K_{p}e-A^{T}\tilde{\lambda }(q,0)=0$.

*Proposition 3.2:* Considering the closed-loop system (2), the equilibrium point (*q*^⋆^, 0) is stable in the sense of Lyapunov given human input (8).

*Proof:* We choose the Lyapunov function as

(9) $$W(q,p)=E+\frac{1}{2}e^{T}K_{p}e-\int_{q_{0}}^{q}A{\left(s\right)}^{T}\tilde{\lambda }(s,0)\cdot \text{d}s-W^{0},$$

where *q*_0_ is the state at *t* = 0 and $W^{0}$ is a constant such that W(q,p) is positive definite and vanishes at the equilibrium point (*q*^⋆^, 0). The integral $\int_{q_{0}}^{q}A{\left(s\right)}^{T}\tilde{\lambda }(s,0)\cdot ds$ is a constant value, where

$$\frac{d}{dt}\left[\int_{q_{0}}^{q}A{\left(s\right)}^{T}\tilde{\lambda }(s,0)\cdot ds\right]=\frac{\partial }{\partial q}\left[\int_{q_{0}}^{q}A{\left(s\right)}^{T}\tilde{\lambda }(s,0)\cdot ds\right]\dot{q}\;={\dot{q}}^{T}A{\left(q\right)}^{T}\tilde{\lambda }(q,0)=0.$$

The Lyapunov function W achieves its minimal point when $\nabla W=0,\text{i.e., }{\left(\partial_{p}W\right)}^{T}={\left(\partial_{p}H\right)}^{T}=M^{-1}p=\dot{q}=0$ and

$${\left(\partial_{q}W\right)}^{T}={\left(\partial_{q}H\right)}^{T}+{\left(\partial_{q}\hat{V}\right)}^{T}+K_{p}e-A^{T}\tilde{\lambda }(q,0)\;=N+{\left(\partial_{q}\hat{V}\right)}^{T}+K_{p}e-A^{T}\tilde{\lambda }(q,0)=0,(\text{with }p=0)$$

i.e., at the equilibrium point (*q*^⋆^, 0). The incorporation of $\int_{q_{0}}^{q}A{\left(s\right)}^{T}\tilde{\lambda }(s,0)\cdot ds$ guarantees the appearance of the GRFs when ∇W(q,0)=0, which coincides with the equilibrium point (*q*^⋆^, 0) at $N+{\left(\partial_{q}\hat{V}\right)}^{T}+K_{p}e-A^{T}\tilde{\lambda }(q,0)=0$. As a result, the Lyapunov function W is positive definite and vanishes only at the equilibrium point (*q*^⋆^, 0).

Applying (8), the time-derivative of Lyapunov function (9) is

W˙=E˙+q˙TKpe−q˙TATλ˜(q,0)=q˙Tτhum +ϕ˙N^3+q˙TKpe=q˙T(−Kpe−Kde˙)+q˙TKpe=−q˙TKdq˙≤0,

which shows that the shaped system is stable in the sense of Lyapunov. ■

Because matrix *K*_*d*_ is only positive semi-definite and $\dot{W}$ does not depend on the full system state, asymptotic stability has not been guaranteed. Proposition 3.2 assumes the human neuromuscular control stabilizes the combined human-exoskeleton system by compensating the moment for global planar orientation. Furthermore, on a trajectory that approaches an equilibrium, our controller will add a bounded amount of energy, where the response of the system will remain in a neighborhood of the equilibrium under human impedance control. This result satisfies our control objective of partial torque assistance while the human controls their kinematics. The human is ultimately responsible for ensuring stability (via impedance control or otherwise), and the passivity property of Proposition 3.1 ensures the stabilization problem is not more difficult with the exoskeleton. Hence, this control approach would not be appropriate for individuals with paraplegia.

## IV. DATA-DRIVEN OPTIMIZATION RESULTS

In [29], we formed multiple basis functions for the shaping terms in (6) and converted our controller design into an optimization process to fit weight-normalized able-bodied joint torque data for variable-incline walking. Although these basis functions aim to change the effect of the gravitational vector and the gyroscopic forces that act within the system, they do not have the flexibility to reproduce joint torques for a broader set of activities, including stair climbing and stand-to-sit. We now re-design the optimization procedure with the incorporation of the global variable *ϕ* and vGRF and validate this procedure with a data-driven simulation. The parameters provided by the optimization are ultimately used in the real-time implementation in Section V.

### A. DESIGN OPTIMIZATION

We design $\hat{N}=-\alpha_{1}\xi_{1}-\cdots -\alpha_{i}\xi_{i}$ and *J*_2_ *M*^−1^*p* = *α*_*i*+1_*ξ*_*i*+1_ + … + *α*_*wξw*_ as linear combinations of the basis functions {*ξ*_1_, *ξ*_2_, …, *ξ*_*w*_} with the constant coefficients $\alpha \in {\mathbb{R}}^{w\times 1}$, where $\xi_{i}\in {\mathbb{R}}^{5\times 1}$ and *w* is the total number of basis functions. We adopt the GRF-based torque tapering strategy from [24] to prevent excess torques during double support, noting that the model (1) does not know the state of the contralateral leg. The vertical GRF (vGRF, which is normalized to one at 100% body weight) and basis functions are incorporated into (6) via

(10) $$u=G^{+}\left(\alpha_{1}\xi_{1}+\alpha_{2}\xi_{2}+\cdots +\alpha_{w}\xi_{w}\right)\cdot \text{vGRF}\;=\Phi (q,p)\alpha \cdot \text{vGRF},$$

where *G*^+^ = [0_2×3_, *I*_2×2_] for the stance leg model and $\Phi (q,p)\in {\mathbb{R}}^{2\times w}$.

We optimize the constant coefficients *α* so the outputs of control law *u* best fit normalized able-bodied human joint torques *y* when inputting able-bodied human kinematic trajectories. The optimization problem is defined as

(11) $$\text{arg }\underset{\alpha }{\text{min}}\sum_{j}\left\{{\left[\text{vGRF}\cdot U\left(q_{j},p_{j},\alpha \right)-Y_{j}\right]}^{T}\cdot W_{j}\left(U,Y_{j}\right)\cdot \left[\text{vGRF}\cdot U\left(q_{j},p_{j},\alpha \right)-Y_{j}\right]+{\left[U^{\text{B}}\left(q_{j},p_{j},\alpha \right)-Y_{j}^{\text{B}}\right]}^{T}W_{k}\left[U^{\text{B}}\left(q_{j},p_{j},\alpha \right)-Y_{j}^{\text{B}}\right]\right\}+U{\left(q_{0},p_{0},\alpha \right)}^{T}W_{r}U\left(q_{0},p_{0},\alpha \right)+\Lambda {\left\|W_{s}\alpha \right\|}_{1},$$

where the subscript *j* represents the number of different walking tasks, including level-ground walking, ramp walking, stair climbing, and stand-to-sit. The state vectors $q_{j},p_{j}\in {\mathbb{R}}^{m\times 1}$ comprise samples over time for the given task *j* with the number of time samples *m*.

The objective function comprises four parts, where the first part corresponds to the least squares error of the exoskeleton control inputs $U\in {\mathbb{R}}^{2m\times 1}$ and the normalized able-bodied human torques $Y_{j}\in {\mathbb{R}}^{2m\times 1}$ for the ankle and knee joints with the weighting matrix *W*_*j*_(*U*, *Y*_*j*_). The weighting matrix *W*_*j*_(*U*, *Y*_*j*_) depends on the exoskeleton and human inputs (*U*, *Y*_*j*_) and adjusts the weights according to sign(*U*(*i*) · *Y*_*j*_(*i*)), where *i* ∈ {1, …, 2*m*} represents the sample index. We enlarge the weights when *U*(*i*) and *Y*_*j*_(*i*) have opposite signs to empha-size the importance of assisting rather than resisting human torques.

The second part of the objective function with *U*^B^ and *Y*^B^ aims to minimize the difference between the control inputs and normalized able-bodied torques during the initial 15% and late 15% of stance phase boundaries with weighting matrix *W*_*k*_, i.e., the early and late stance phases during the gait cycle, without the effect of GRFs. This helps regulate the exoskeleton torques *u* at endpoints of the stance phase. This also minimizes the dependence on vGRF for real-time implementation to avoid aggressive torques when the custom force sensor in [24] returns inconsistent measurements of vGRF compared to the force plates in the dataset [33].

We also include *W*_*r*_ with states *p* = *p*_0_ = 0 and *q* = *q*_0_ in the third part of the objective function, where *q*_0_ is the state when *ϕ*, *θ*_*a*_ = 0 and *θ*_*k*_ is hyper-extended. This encourages the optimization to provide minimal knee torque during hyper-extension for safety. Lastly, similar to [34], we apply “L1 regularization” to enforce sparsity in the model by zeroing the least important parameters in vector *α*, which avoids over-fitting and improves the prediction of untrained tasks. The term Λ weights the penalty on the number of basis functions. The weighting matrix *W*_*s*_ adjusts the optimal parameters *α* to focus more on shaping the gyroscopic terms or the modified potential energy. We use “fmin-con” with sequential quadratic programming in Matlab to find the optimal solution α*. The corresponding control law equals *u* = Φ(*q*, *p*) α* · vGRF · LOA%, where LOA% (level-of-assistance) scales down the controller to a desired fraction of normative torque.

We compare two shaping strategies: 1) Hamiltonian without *ϕ* (WOP) has basis functions depending on *θ*_*a*_ and *θ*_*k*_ only, and 2) Hamiltonian with *ϕ* (PHI) has the global variable *ϕ* incorporated into the basis functions. To begin, we defined 67 basis functions in *ξ*_ankle_, $\xi_{\text{knee}}\in {\mathbb{R}}^{67\times 1}$ in the form of gyroscopic or potential forces, where the total number was determined empirically (see Supplementary Material). The PHI method uses all 67 basis functions, whereas the WOP method removes all terms depending on *ϕ* (satisfying [29, Proposition 1]) for a total of 35 basis functions. Both cases have $\Phi (q,p)={\left[\xi_{\text{ankle}},\xi_{\text{knee}}\right]}^{T}\in {\mathbb{R}}^{2\times w}$ in (10). Column vectors in Φ(*q*, *p*) associated with the shaped gyroscopic terms are orthogonal to ${\left[{\dot{\theta }}_{a},{\dot{\theta }}_{k}\right]}^{T}$. In contrast, column vectors in Φ(*q*, *p*) associated with the shaped potential energy introduce conservative forces corresponding to modified gravity and nonlinear virtual springs.

We optimize the constant coefficients *α* to fit the control law outputs to normalized values of the across-subject (ten subjects) averaged human joint torques over level-ground, ramps, stairs walking [33], and stand-to-sit [35]. The optimization process provides the optimal parameters α*, where we neglect those parameters with absolute values contributing less than 0.1% ∥α*∥_2_. The vGRFs during locomotion tasks in [33] are normalized by the body weight. Because the stand-to-sit data in [35] does not provide vGRFs, we set the vGRF to a constant value during the optimization process (a reasonable assumption for a quasi-static task like stand-to-sit). The training tasks include level treadmill walking at 0.5, 1.5m/s, ascending/descending ramps with inclines of 5.2°, 11°, ascending/descending stairs with step height of 4, 7inch [33], and the stand-to-sit task in [35]. The original knee torque data in [35] was not adequate during the late stand-to-sit cycle because of support from the chair during data collection. To provide adequate support with our controller, we modified the able-bodied knee torque profile from [35] by holding the peak knee torque from 60% to 100% of the cycle.

**FIGURE 3.**
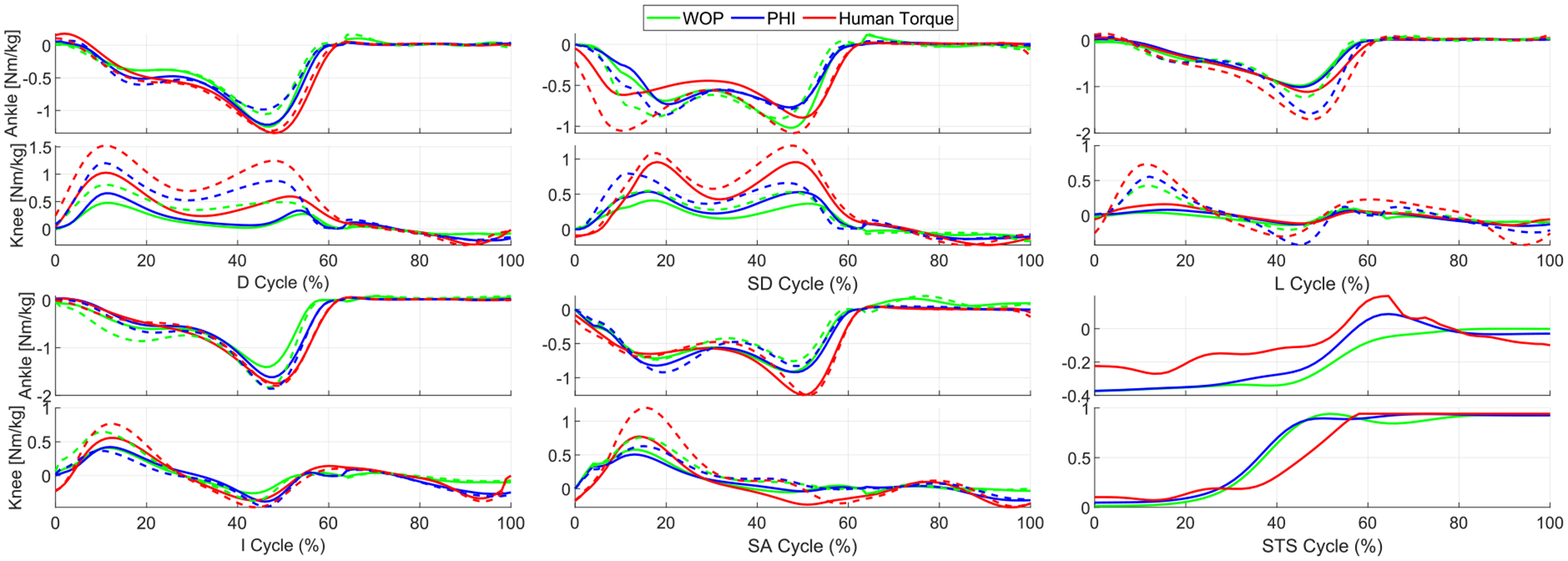
Estimated exoskeleton control torques and weight-normalized able-bodied human torques based on human treadmill walking (L) at 0.5m/s (solid lines) and 1.5m/s (dash lines), ramp ascent/descent (I/D) at 5.2° (solid lines) and 11° (dash lines), stairs ascent/descent (SA/SD) on 4inch (solid lines) and 7inch (dash lines) steps, and stand-to-sit (STS). Positive values represent ankle dorsiflexion and knee extension.

Fig. 3 shows the simulated exoskeleton control torques and demonstrates the agreement between a single energy-shaping control strategy (exoskeleton torque *τ*_exo_) and normalized able-bodied human torques *τ*_hum_ over the training activities. The validation activities are considered next in comparison with a state-of-art finite state machine (FSM) controller [5].

### B. COMPARISON TO IDEAL FINITE STATE MACHINE

The presented method was evaluated by comparison with an ideal FSM for testing tasks. We defined the FSM in a similar way as in [36], where the ideal FSM was assumed to provide the normalized able-bodied human torque with pre-defined tasks using intent recognition between different modes, including walking and stairs climbing. The pre-defined “training” tasks included level treadmill walking at 1.5 m/s, ramp ascent/descent at 5.2°, and stairs ascent/descent with 4inch step height in [33] to cover a similar number of tasks to a state-of-art FSM [5]. The ideal FSM returns the pre-defined torque profile *Y*_*j*_, *j* ∈ {1, …, 5}, that most closely matches the normalized able-bodied profile *Y*_*i*_ for the current task *i*. The problem is defined in [36, 7] as finding *j* in the pre-defined tasks via

$$\text{arg }\underset{j}{\text{min}}{\left\|Y_{i,\text{ankle}}-Y_{j,\text{ankle}}\right\|}_{2}+{\left\|Y_{i,\text{knee}}-Y_{j,\text{knee}}\right\|}_{2}\text{. }$$

Although this FSM is difficult to implement in practice (specifically real-time classification of the nearest task [5]), it provides a useful standard of comparison representing the minimum possible error with the FSM approach [36].

We used two metrics for comparison of the energy-shaping and FSM methods. The first metric used was a Cosine Similarity (SIM), which is a judgment of orientation that measures the pattern of the normalized able-bodied torques. The second metric used was the Variance Accounted For (VAF) which measures the variability of the data that can be explained by a fitted regression model. The definitions are

$$\text{SIM}(A,B)=\frac{100\cdot A\cdot B}{\|A{\|}_{2}\|B{\|}_{2}},$$

$$\text{VAF}(A,B)=100\left(1-\frac{\text{variance}(A-B)}{\text{variance}(A)}\right).$$

We measured the metrics on knee and ankle torques separately and averaged them together for a single quantity.

We performed leave-one-subject-out (ten subjects in total) cross-validation to check the predictive performance of the proposed methods in the presence of subject-specific variations in joint torque. The validation tasks included all the training tasks in Section IV-A and additional tasks of level treadmill walking at 0.65 m/s, ascending/descending a 9.2° ramp, and ascending/descending stairs with 6inch step height [33]. To compare the different methods, we performed group statistics (*n* = 10) on the SIM and VAF scores calculated from each subject’s joint torques and the predicted torques from the corresponding model trained without that subject’s data. Since SIM and VAF were not normally distributed (according to the Shapiro Wilk test for normality), we applied a non-parametric test for checking the statistical significance of the effect of control method (PHI, WOP, FSM) on SIM and VAF. For each task, we performed pairwise comparisons between methods using the Wilcoxon signed-rank test with the null hypothesis that the median difference in score between the different modes was zero.

**FIGURE 4.**
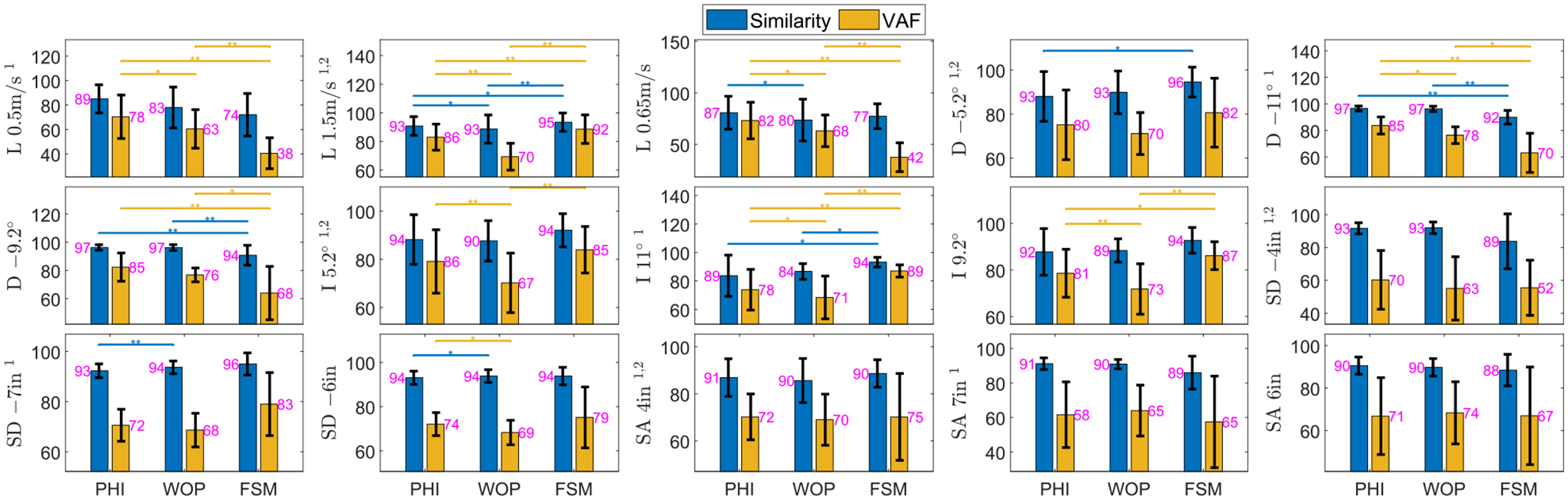
Comparison of techniques with metrics averaged over the knee and ankle. L, D/I, and SD/SA denote level walk, decline/incline walk, and stair descent/ascent, respectively. 1 denotes the tasks in the training process of WOP and PHI methods, while 2 denotes the pre-defined “training” tasks in FSM. Stand-to-sit task is not included in the leave-one-subject-out cross-validation due to the lack of data with multiple subjects in [35]. Number in pink denotes the median. * represents statistical difference (*p* < 0.05). ** represents *p* <= 0.01. *** represents *p* <= 0.001.

As shown in Fig. 4, both the PHI and WOP methods performed well with different tasks under both metrics, with minor advantages for the PHI method. Averaged over all tasks and subjects, PHI has mean SIM = 89.5 ± 9.2% and VAF = 73.4 ± 14.7%. WOP has mean SIM = 88.7 ± 10.3% and VAF = 68.1 ± 12.8%. FSM has mean SIM = 88.8 ± 10.7% and VAF = 69.0 ± 21.6%. The method in [29] (not shown in Fig. 4) has mean SIM = 81.0 ± 9.2% and VAF = 53.8 ± 14.9%, which are much lower than all other methods. Overall PHI has higher mean SIM and VAF compared to FSM, with this trend being statistically significant at the significance level 0.05 in 2 out of 15 tasks for SIM score and 4 out of 15 tasks for VAF score. The FSM method significantly outperformed the energy-shaping methods for ramp ascent because joint torques do not change much between different ramp inclines, i.e., the data of {9.2, 11}° matched closely to the FSM training tasks. Although the medians were different in stair ascent and descent cases, the difference did not reach the significance level 0.05. For testing tasks that do not closely resemble any pre-defined tasks, the FSM performance drops substantially, e.g., {−9.2, −11}° ramp descent and level walking at {0.5, 0.65} m/s. In fact, the FSM’s worst performance with median VAF = 38% during level walking at 0.5 m/s is much worse than PHI’s worst performance with median VAF = 58% in stair ascent 7inch. In practice, the FSM method would not be able to use future trajectory information to achieve ideal task classification, resulting in substantially worse errors when choosing the wrong controller.

The energy-shaping controller can be improved by retraining with all tasks (including testing data), but the FSM is always limited to one condition per activity. For consistency, the experimental implementation in Section V uses the average subject’s optimization results presented in Fig. 3 (without re-training).

## V. EXPERIMENTAL VALIDATION WITH HUMAN SUBJECTS

In this section, we implement the controller on a backdrivable knee-ankle exoskeleton and use it to partially assist multiple healthy human subjects performing multiple ADLs. The control torques and resulting muscle activation demonstrate the versatility of the proposed control approach in providing biomimetic assistance across multiple activities.

### A. HARDWARE IMPLEMENTATION

The controller was implemented on the *Comex* knee-ankle exoskeleton shown in Fig. 1 (see [10], [24] for details). *Comex* weighs 4.5 kg, and has backdrivable actuators due to their low 24:1 gear ratio. Both knee and ankle modules produce 30 Nm continuous torque (60 Nm peak) using 200 W frameless BLDC motors. The high-level control loop runs at 500 Hz on a National Instruments MyRIO. Joint angle feedback is provided by high-resolution magnetic incremental encoders, and a 6-axis inertial measurement unit provides the global thigh orientation. *Comex* is powered by a 24 V Li-Ion battery housed inside a backpack. Safety features such as hard stops and current limiters are present at both joints. See Supplementary Figure S1 which illustrates the attachments and adjustments of *Comex*.

The vGRF is measured by a custom force sensor attached to the bottom of *Comex’s* footplate. Force is measured by multiple force-sensitive resistors (FSRs), which are sandwiched between two rigid plates held apart by circular pucks. The design was inspired by force plates. The sensor is calibrated before each use to achieve a final readout normalized to body weight in the same manner as the vGRFs from the normative dataset used for the controller simulation. The final values of vGRFs are saturated within [0,1] on the MyRIO to avoid excessive assistance torques.

Although the *Comex* actuators are backdrivable [10], the ankle backdrive torque is still significant compared to normative ankle dorsiflexion torques during the swing phase of gait (around 5 Nm). The active modes in [29] did not reduce muscle activation of tibialis anterior, where the assistive dorsiflexion torques in the swing phase (>60% stride) were lower than the estimated backdrive torque (3 Nm, see [10, Fig. 16]). This suggests the subject experienced more resistance than assistance. To reduce the backdrive torque acting on the ankle joint without the use of torque sensors, we adopt the inertia compensation methodology described in [37]. The torques induced by inertia are determined by $\tau_{\text{inertia}}=\ddot{\Theta }\cdot I_{\text{reflected}}$, where $\ddot{\Theta }$ represents the angular acceleration. The reflected inertia is approximated by the product of rotor inertia and gear ratio squared [11]. For *Comex*, the reflected inertia *I*_reflected_ = 691.5 kg-cm^2^. We apply inertia compensation to the ankle when ${\ddot{\theta }}_{a}\geq 0$ to assist dorsiflexion and avoid torque oscillation around ${\ddot{\theta }}_{a}=0$. We also saturate the inertia compensation within [0,2.5] Nm. Therefore, the resulting inertia compensation term is given by

$$\tau_{\text{inertia,ankle}}=\{\begin{array}{ll} {\text{sat}}_{\geq 0}^{\leq 2.5}\left({\ddot{\theta }}_{a}\cdot I_{\text{reflected}}\right), & \text{if }{\ddot{\theta }}_{a}\geq 0\\ 0, & \text{otherwise} \end{array}$$

Since the control law provides small dorsiflexion torques in Fig. 3, we also amplify the optimal control input *u*_opt, ankle_ when the assistive dorsiflexion torques are lower than the estimated backdrive torque (3 Nm). A scaling value of 1.3 was chosen based on the comfort level of our pilot subject. For dorsiflexion torques higher than the estimated backdrive torque, the optimal control input *u*_opt, ankle_ remains unchanged. Incorporating these features, the control input for the ankle joint is given by

$$\tau_{\text{ankle}}=\{\begin{array}{ll} 1.3\cdot u_{\text{opt},\text{ ankle}}+\tau_{\text{inertia, ankle}}, & \text{if }u_{\text{opt},\text{ ankle}}\in [0,3]\\ u_{\text{opt},\text{ ankle}}+\tau_{\text{inertia, ankle}}, & \text{otherwise } \end{array}$$

where *u*_opt, ankle_ represents (10) in Nm/kg multiplied by the subject’s body mass and LOA%. The knee control input does not include the inertia compensation features. Before conducting the human subject study, we adjusted the weighting factors in the optimization process (11) for user comfort during several practice trials (see Supplementary Material).

### B. HUMAN SUBJECT METHODS

The following study was approved by the Institutional Review Board at the University of Michigan (HUM00164931). We enrolled five able-bodied human subjects (s1, male, mass: 78 kg, height: 1.78 m; s2, male, mass:75 kg, height: 1.75 m; s3, female, mass: 50 kg, height: 1.62 m; s4, male, mass: 83 kg, height: 1.79 m; s5, female, mass: 60 kg, height: 1.75 m) to demonstrate the controller’s ability to assist multiple tasks. Two subjects (s4, s5) were excluded due to failure of a foot FSR causing unusual control torques, which was noticed after the experiment. The remaining subjects had substantial (s1), moderate (s2), or minimal (s3) experience with *Comex*. We assessed muscle activation via EMG (Delsys Inc.) of vastus medialis oblique (VMO), rectus femoris (RF), biceps femoris (BF), tibialis anterior (TA), gastrocnemius (GM), and soleus (SOL), which function as a knee extensor, knee extensor/hip flexor, knee flexor, dorsiflexor, plantarflexor/knee flexor, and plantarflexor respectively. See Supplementary Figure S2 for EMG electrode placement.

The experiment comprised level treadmill walking at self-selected speed (1 m/s for s1–2, 0.8 m/s for s3), incline/decline treadmill walking on a ±5.2° slope at 0.6 m/s and a ±12.4° slope at 0.6 m/s, repetitive sit-stand cycles with a metronome set to 45 beats-per-minute (BPM), and stairs ascent/descent over 7 inch steps with a 60 BPM metronome. The tasks were repeated for three exoskeleton modes: bare (no exoskeleton), active exoskeleton with *ϕ* (PHI), and active exoskeleton without *ϕ* (WOP). The LOA% for the active modes was set to 60% for s1 and 50% for other subjects, based on their comfort level during practice trials. We collected at least 30 gait cycles for each treadmill task, 18 gait cycles for each stair task, and 18 sit-stand cycles. Subjects were instructed not to use the treadmill handrails except to prevent a fall (which never occurred). A supplementary video of the experiments is available for download.

The walking trials were cropped into gait cycles by detecting heelstrike with a heel-mounted accelerometer. Sit-stand-sit trials were cropped into individual repetitions using a thigh-mounted accelerometer built into the EMG sensor. Each muscle’s EMG was demeaned, bandpass filtered (20 – 200 Hz), smoothed with a moving 100 ms window RMS, and then normalized with respect to the maximum peak of the ensemble averages (across repetitions) of the three exoskeleton modes [38]. This was done for each task and muscle separately, resulting in the signals being converted to a percentage of the maximum voluntary contraction level (%MVC) for a consistent and fair comparison across subjects. After normalizing the EMG to %MVC, the integral with respect to time was calculated to represent muscular effort as %MVC.s, similar to [24].

We performed intra-subject statistics on the EMG effort data. Since these data were not normally distributed according to the Shapiro Wilk test for normality, we applied non-parametric tests for checking the statistical significance of the effect of controller mode on EMG effort for each subject, similar to [39]. We first used the Friedman’s test to check the null hypothesis that muscle effort data corresponding to the three modes came from the same population. When the null hypothesis was rejected (*α* = 0.05), we performed post-hoc pairwise comparisons between modes using the Wilcoxon signed-rank test with the null hypothesis that no median difference existed between EMG effort from different modes.

### C. HUMAN SUBJECT RESULTS

**FIGURE 5.**
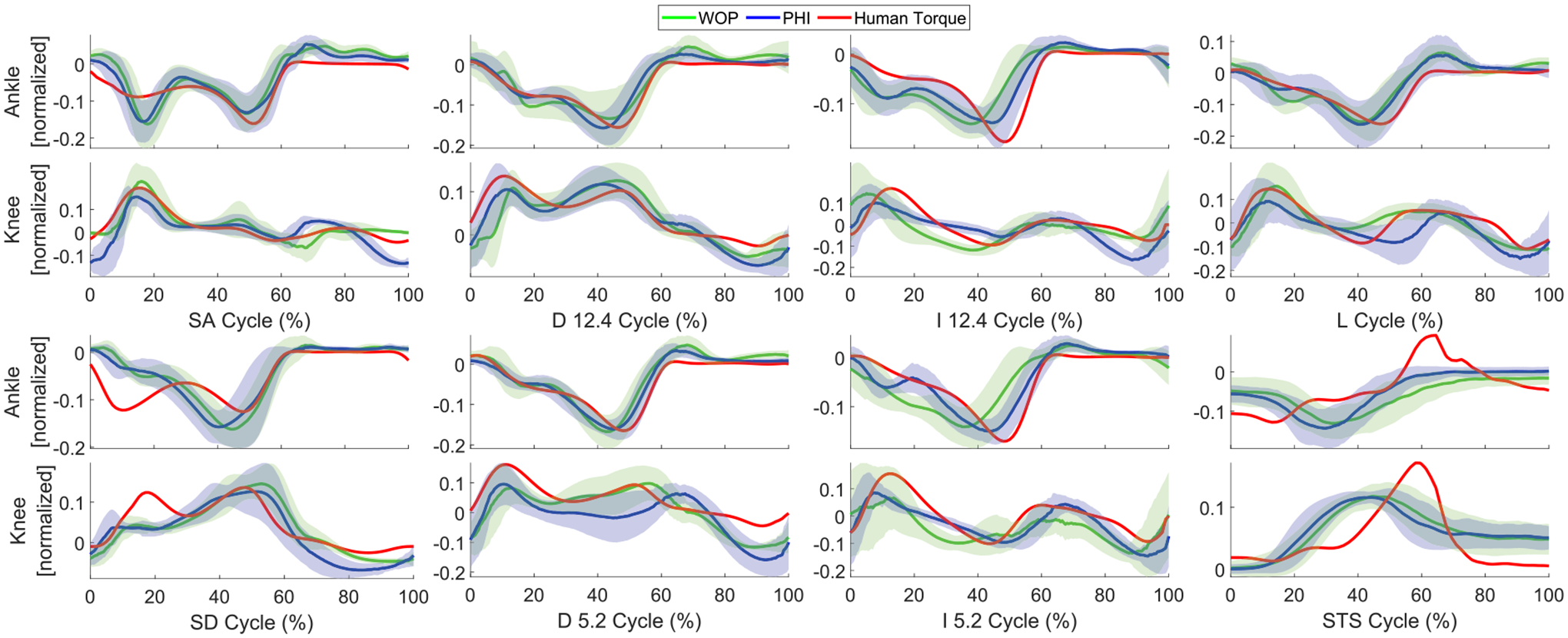
Comparisons of across-subject averaged normalized command torques (PHI and WOP methods) and normalized able-bodied human torques for experiment tasks {stair ascent/descent (7inch), decline (−5.2°, −12.4°) and incline (5.2°, 12.4°), level ground (1 m/s), stand-to-sit}. The blue solid (PHI method) and green solid (WOP method) lines represent the mean commanded exoskeleton torque (normalized by L2 norm) across all repetitions for the active modes. The red solid line represents the normative human joint torques (normalized by L2 norm) in [33], [35]. Positive torques represent ankle dorsiflexion and knee extension.

Fig. 5 shows that, even in the experiment with subject kinematics being influenced by the exoskeleton’s mass and joint torque, the averaged command torques (PHI and WOP methods) match with the normalized able-bodied human torques from [33], [35] in most tasks with SIM = 81.6 ± 6.5%,VAF = 60.4 ± 16.3% for PHI and SIM = 80.1 ± 9.0%,VAF = 50.8 ± 19.2% for WOP, where torque trajectories are normalized to the L2 norm and standard deviations are given over tasks. The mismatch was likely due to multiple factors. Firstly, there may be a mismatch between reference kinematics from literature and the feedback joint angles and IMU information due to compliance in straps, padding, and soft tissue. Individual variations in kinematics, as well as variations in the individual responses to the assistive torques could also explain the mismatch. In addition, the vGRFs were measured by the custom force sensor in the *Comex* footplate and saturated between [0,1], which gives slightly different values compared to a force plate.

**FIGURE 6.**
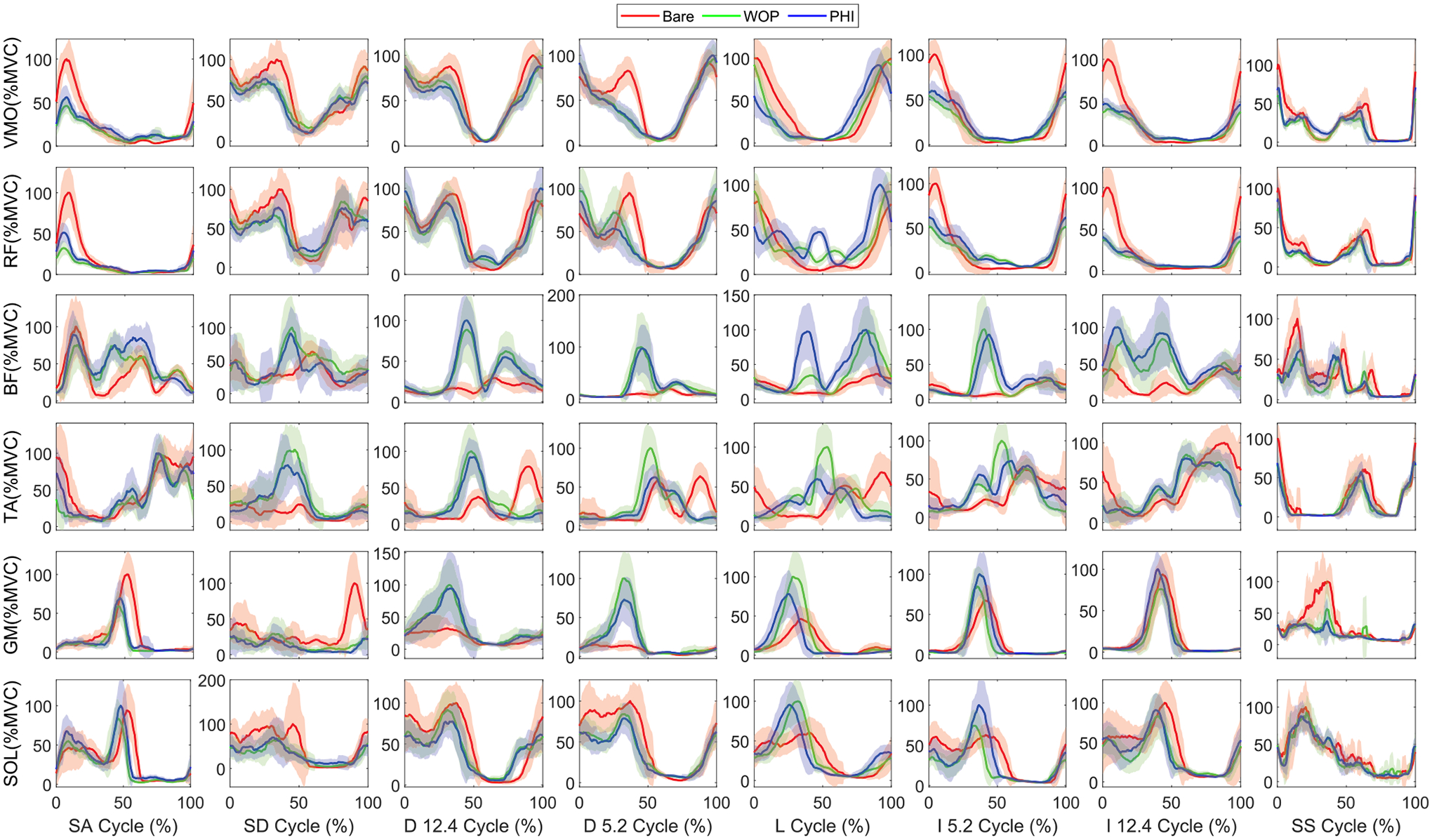
Subject 1 EMG comparisons between bare and active modes (PHI and WOP methods) for each muscle (VMO, RF, BF, TA, GM and SOL) and task {Stairs Ascent/Descent (7 in step height), Decline (−5.2°, −12.4°) at 0.6 m/s, level ground (1 m/s), Incline (5.2°, 12.4°) at 0.6 m/s, and Sit-Stand cycle (45 BPM)}. The red solid (bare), blue solid (PHI method), and green solid (WOP method) lines represent the time-normalized ensemble averages across all repetitions.

**FIGURE 7.**
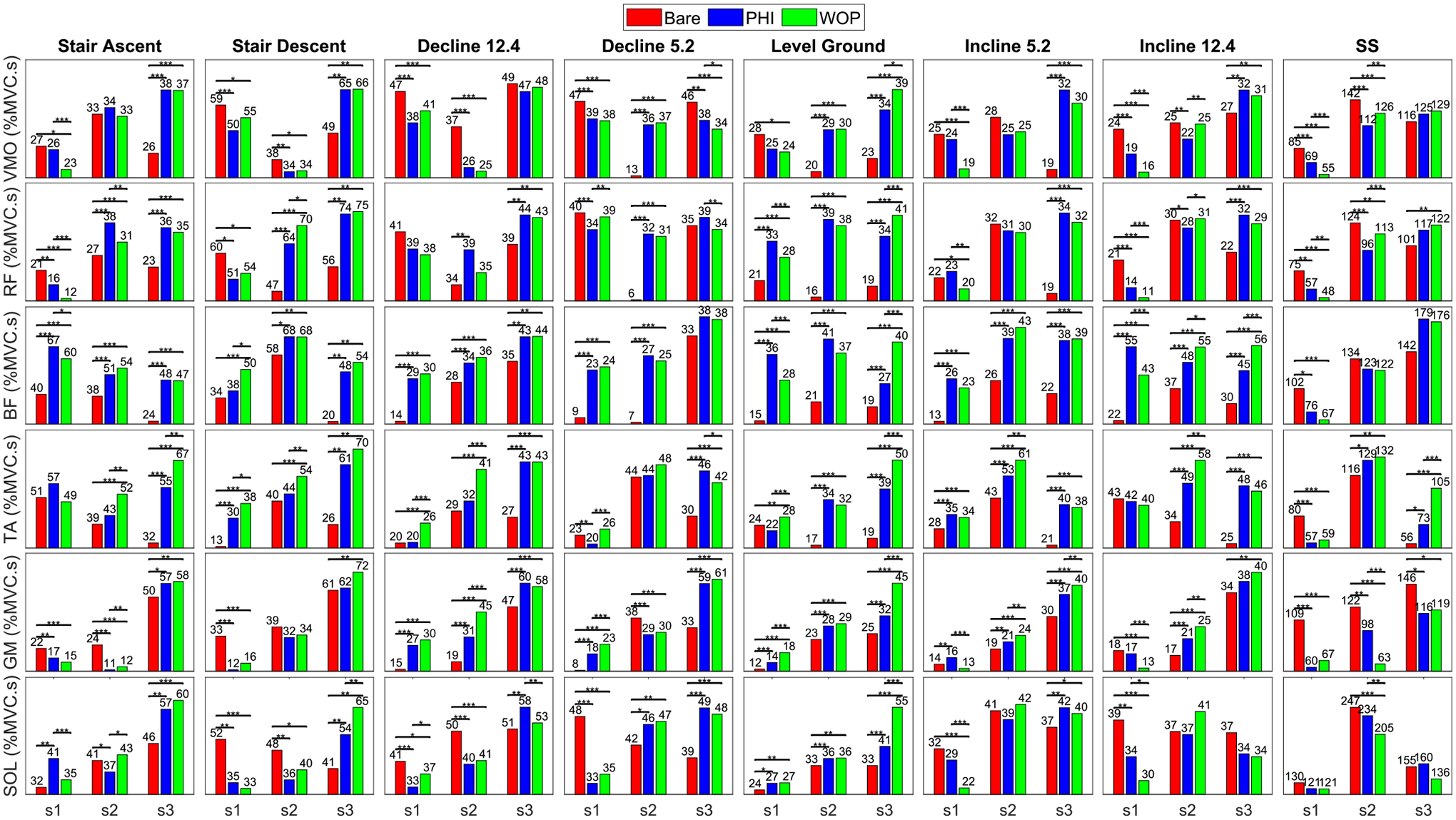
Individual subject comparisons of mean effort across repetitions. Effort is compared between bare, active with PHI method, and active with WOP method for each muscle pair (VMO, RF, BF, TA, GM and SOL) and task {Stairs Ascent/Descent (7 in step height), Decline (−5.2°, −12.4°) at 0.6 m/s, level ground (1 m/s for s1 and s2, 0.8 m/s for s3), Incline (5.2°, 12.4°) at 0.6 m/s, and Sit-Stand cycle (45 BPM)}. * represents statistical difference (*p* < 0.05). ** represents *p* <= 0.01. *** represents *p* <= 0.001.

The ensemble-averaged VMO, RF, BF, TA, GM, and SOL EMGs for bare and active modes are shown in Fig. 6 for s1, who was the best responding subject to exoskeleton assistance. In general the task-specific dominant muscles (for the stance phase) had reduced effort and peak EMG for the active modes in most tasks—VMO, GM, and SOL for treadmill and stairs tasks, and VMO for sit-stand. Fig. 7 quantifies this trend for EMG effort and provides intra-subject statistics for the various muscles and tasks. Moreover, the assistance torque profiles matched the muscle activation profiles, explaining the reduction in muscle activation compared to bare mode. See Supplementary Figures S3–S7 for individual subject EMG ensemble averages, across-subject ensemble averages, across-subject effort and peak EMG plots, and photos of the different task experiments, respectively.

Incline walking and stairs ascent are primarily associated with positive power or concentric muscle contractions. In these tasks, the quadriceps are predominantly activated to lift the center of mass (COM) of the body. Both PHI and WOP provided knee extension torques in this phase and resulted in EMG reduction of the VMO for s1 and s2. Both controllers provided plantar-flexion torques in this phase for stairs ascent and incline walking, resulting in noticeable GM and SOL EMG reductions compared to the bare mode for s1 and s2 with stairs ascent. For s3, there was only a noticeable reduction in this phase for SOL with incline walking.

Stairs descent and decline walking are primarily associated with negative power and involve eccentric quadriceps and plantar-flexor contractions. Commonly, a double peak quadriceps activation profile is apparent in stance; firstly to absorb the impact of heel strike, and secondly to lower the COM. Both controllers provided knee extension torques during these phases, which resulted in substantial EMG reductions compared to the bare mode of the VMO for s1 with all stairs descent and decline walking tasks, and s2 and s3 with most stairs descent and decline walking tasks. Both controllers provided substantial plantar-flexion assistance torques during mid to late stance to assist with the negative work of lowering the COM. This resulted in substantial reductions in SOL activity compared to the bare mode for s1 with all stairs descent and decline walking tasks, and s2 with most stairs descent and decline walking tasks. Note that the SOL is more active during flexed knee positions (such as decline walking or stairs descent) than GM, which is more active during extended knee positions.

Sit-to-stand and stand-to-sit primarily require knee extension torques [40]. These occur in the form of concentric contractions during sit-to-stand and eccentric contractions during stand-to-sit. Both controllers provided substantial knee extension torques, resulting in a noticeable reduction in VMO (knee extensor) activations for s1 and s2. Results of GM and SOL had high inter-subject variability due to the low muscle activation in the sit-stand cycle compared to the dominant muscles (VMO and RF).

Lastly, we observed some reductions in the quadriceps and the plantar-flexors during the stance phase of level walking. The quadriceps have a high activation in bare mode primarily to dampen the impact of heel strike. The plantar-flexors provide the pushoff power during late stance to drive the COM forwards. Both our controllers provided appropriate knee extension and plantar-flexion assistance torques that resulted in noticeable reductions in VMO (s1) and SOL (s2) activity in the stance phase. Since the knee goes through a minimal range of motion during stance in level walking, our prior controller that utilized only potential energy shaping [24] was not adequate to provide assistance during this phase. With the PHI and WOP controllers developed in the present study, adequate knee extension assistance torques are provided to assist with impact absorption in early stance.

The TA activations for both PHI and WOP were higher than bare for all walking tasks. This is similar to the results in [41], where the TA during the swing phase had increased activity with decreasing gravity. One explanation is that we are not providing adequate torques to support the weight of the sensorized exoskeleton foot plate. It is also possible that the provided plantar-flexion torques are excessive, necessitating the TA activation to compensate. Future work will model the passive dynamics of the muscle-tendon unit for all joints. This is especially important for the ankle, i.e., the Achilles tendon is known to provide significant storage and release of energy, much like a spring.

The purpose of BF during swing is to lift the foot by flexing the knee, aiding in leg clearance. Although we provided marginal knee flexion torques, we observed high activations for BF with the active modes compared to bare, which was also found in [41] during stance phase. A potential explanation can be the interaction with its second function as a hip extensor and needing to carry the added weight of the exoskeleton during swing, which can also affect RF.

Figs. 6 and 7 demonstrate the potential to assist musculature across multiple tasks. Note that each EMG signal is normalized as %MVC with respect to the maximum peak of the ensemble averages, which does not reflect the differences between dominant and non-dominant muscles for each task. For instance, during decline walking (−12.4°), VMO is dominant and has a large reduction in EMG with active modes, whereas the non-dominant BF has the opposite effect. We believe that improvement in dominant muscles carries more weight than worsening of non-dominant muscles when assessing the overall performance of the proposed methods.

The subject-wise muscular efforts in Fig. 7 demonstrate that s1 and s2 responded better to orthosis assistance than s3 for some muscles and tasks (see also Fig. 6 and Supplementary Material). This could be due to the fact that s3 was relatively short and lightweight compared to the large exoskeleton used in this study, or due to the inexperience of s3. We provided the subjects with approximately 2 minutes of acclimation time for each task, whereas a prior study gave 30 minutes of acclimation time before showing EMG reductions under the assistance [42]. It is thus possible that our outcomes would improve by providing more acclimation time. Additional human subjects would be needed to draw more general conclusions about the controller’s effectiveness, which is left to future work.

## VI. CONCLUSION

This paper applied a novel energetic control strategy based IDA-PBC that can assist all primary ADLs with a backdrivable knee-ankle exoskeleton. Whereas prior work on passivity-based energy-shaping control behaved as nonlinear virtual springs, this paper incorporated global orientation and vGRF feedback to broaden the capabilities of the controller while preserving input-output passivity and stability of the closed-loop system. We increased the candidate basis functions in the optimization process, which achieved an optimal controller that fits normalized able-bodied human joint torques more closely for more tasks. We considered “L1 regularization,” which fits the data with as few parameters as possible to avoid overfitting problems. We also demonstrated the potential of the implemented controller to reduce muscular effort in a human subjects study involving level-ground, ramp, and stairs walking as well as sit-stand transitions.

Future work could consider inconsistencies between the optimization dataset and real-time GRF data from exoskeleton sensors. Moreover, lighter backdrivable exoskeletons are being developed [11], [12] that could avoid co-contractions and/or compensations associated with exoskeleton mass, enabling more consistent reductions in muscle activation. Future work could also incorporate the passive and active dynamics of the relevant muscle-tendon units to further improve biomimicry of the assistance torque.

## Supplementary Material

supplemental pdf

supplemental video
