# Supplementary material for: Optimally Biomimetic Passivity-Based Control of a Lower-Limb Exoskeleton Over the Primary Activities of Daily Life
Source: IEEE Open J Control Syst. Author manuscript; Available in PMC 2022 Jun 6. (PMC9170045)
Supplement: supplemental pdf [file NIHMS1805282-supplement-supplemental_pdf.pdf]

Optimally Biomimetic Passivity-Based Control of a Lower-Limb Exoskeleton over the Primary Activities of Daily Life: supplemental material

1. DESIGNED BASIS FUNCTIONS

PHI has ϕ incorporated with 67 basis functions, where the list of basis functions for the ankle and the knee joints $\xi_{\text{ankle}}, \xi_{\text{knee}} \in \mathbb{R}^{67\times 1}$ are shown as follows

$$
\begin{aligned}
\xi_{\text{ankle}} = [&1, 0, \sin(\theta_a), \cos(\theta_a), \dots \\
&0, 0, \sin(\theta_a+\theta_k), \cos(\theta_a+\theta_k), \dots \\
&\sin(2\theta_a), \cos(2\theta_a), 0, 0, \dots \\
&\sin(2\theta_a+2\theta_k), \cos(2\theta_a+2\theta_k), 2\sin(2\theta_a+\theta_k), 2\cos(2\theta_a+\theta_k), \dots \\
&\sin(\theta_a+2\theta_k), \cos(\theta_a+2\theta_k), \sin(\phi+\theta_a), \cos(\phi+\theta_a), \dots \\
&0, 0, \sin(\phi+\theta_a+\theta_k), \cos(\phi+\theta_a+\theta_k), \dots \\
&\sin(\phi+2\theta_a), \cos(\phi+2\theta_a), 0, 0, \dots \\
&\sin(\phi+2\theta_a+2\theta_k), \cos(\phi+2\theta_a+2\theta_k), 2\sin(\phi+2\theta_a+\theta_k), 2\cos(\phi+2\theta_a+\theta_k), \dots \\
&\sin(\phi+\theta_a+2\theta_k), \cos(\phi+\theta_a+2\theta_k), \dot{\theta}_k, \dots \\
&\sin(\theta_a)\dot{\theta}_k, \cos(\theta_a)\dot{\theta}_k, \sin(\theta_k)\dot{\theta}_k, \cos(\theta_k)\dot{\theta}_k, \dots \\
&\sin(\theta_a+\theta_k)\dot{\theta}_k, \cos(\theta_a+\theta_k)\dot{\theta}_k, \sin(2\theta_a)\dot{\theta}_k, \cos(2\theta_a)\dot{\theta}_k, \dots \\
&\sin(2\theta_k)\dot{\theta}_k, \cos(2\theta_k)\dot{\theta}_k, \sin(2\theta_a+2\theta_k)\dot{\theta}_k, \cos(2\theta_a+2\theta_k)\dot{\theta}_k, \dots \\
&\sin(2\theta_a+\theta_k)\dot{\theta}_k, \cos(2\theta_a+\theta_k)\dot{\theta}_k, \sin(\theta_a+2\theta_k)\dot{\theta}_k, \cos(\theta_a+2\theta_k)\dot{\theta}_k, \dots \\
&\sin(\phi+\theta_a)\dot{\theta}_k, \cos(\phi+\theta_a)\dot{\theta}_k, \sin(\phi+\theta_k)\dot{\theta}_k, \cos(\phi+\theta_k)\dot{\theta}_k, \dots \\
&\sin(\phi+\theta_a+\theta_k)\dot{\theta}_k, \cos(\phi+\theta_a+\theta_k)\dot{\theta}_k, \sin(\phi+2\theta_a)\dot{\theta}_k, \cos(\phi+2\theta_a)\dot{\theta}_k, \dots \\
&\sin(\phi+2\theta_k)\dot{\theta}_k, \cos(\phi+2\theta_k)\dot{\theta}_k, \sin(\phi+2\theta_a+2\theta_k)\dot{\theta}_k, \cos(\phi+2\theta_a+2\theta_k)\dot{\theta}_k, \dots \\
&\sin(\phi+2\theta_a+\theta_k)\dot{\theta}_k, \cos(\phi+2\theta_a+\theta_k)\dot{\theta}_k, \dots \\
&\sin(\phi+\theta_a+2\theta_k)\dot{\theta}_k, \cos(\phi+\theta_a+2\theta_k)\dot{\theta}_k]^T,
\end{aligned}
$$

$$
\begin{aligned}
\xi_{\text{knee}} = [&0, 1, 0, 0, \dots \\
&\sin(\theta_k), \cos(\theta_k), \sin(\theta_a+\theta_k), \cos(\theta_a+\theta_k), \dots \\
&0, 0, \sin(2\theta_k), \cos(2\theta_k), \dots \\
&\sin(2\theta_a+2\theta_k), \cos(2\theta_a+2\theta_k), \sin(2\theta_a+\theta_k), \cos(2\theta_a+\theta_k), \dots \\
&2\sin(\theta_a+2\theta_k), 2\cos(\theta_a+2\theta_k), 0, 0, \dots \\
&\sin(\phi+\theta_k), \cos(\phi+\theta_k), \sin(\phi+\theta_a+\theta_k), \cos(\phi+\theta_a+\theta_k), \dots \\
&0, 0, \sin(\phi+2\theta_k), \cos(\phi+2\theta_k), \dots \\
&\sin(\phi+2\theta_a+2\theta_k), \cos(\phi+2\theta_a+2\theta_k), \sin(\phi+2\theta_a+\theta_k), \cos(\phi+2\theta_a+\theta_k), \dots \\
&2\sin(\phi+\theta_a+2\theta_k), 2\cos(\phi+\theta_a+2\theta_k), -\dot{\theta}_a, \dots
\end{aligned}
$$

$$
\begin{aligned}
&-\sin(\theta_a)\dot{\theta}_a, -\cos(\theta_a)\dot{\theta}_a, -\sin(\theta_k)\dot{\theta}_a, -\cos(\theta_k)\dot{\theta}_a, \ldots \\
&-\sin(\theta_a+\theta_k)\dot{\theta}_a, -\cos(\theta_a+\theta_k)\dot{\theta}_a, -\sin(2\theta_a)\dot{\theta}_a, -\cos(2\theta_a)\dot{\theta}_a, \ldots \\
&-\sin(2\theta_k)\dot{\theta}_a, -\cos(2\theta_k)\dot{\theta}_a, -\sin(2\theta_a+2\theta_k)\dot{\theta}_a, -\cos(2\theta_a+2\theta_k)\dot{\theta}_a, \ldots \\
&-\sin(2\theta_a+\theta_k)\dot{\theta}_a, -\cos(2\theta_a+\theta_k)\dot{\theta}_a, -\sin(\theta_a+2\theta_k)\dot{\theta}_a, -\cos(\theta_a+2\theta_k)\dot{\theta}_a, \ldots \\
&-\sin(\phi+\theta_a)\dot{\theta}_a, -\cos(\phi+\theta_a)\dot{\theta}_a, -\sin(\phi+\theta_k)\dot{\theta}_a, -\cos(\phi+\theta_k)\dot{\theta}_a, \ldots \\
&-\sin(\phi+\theta_a+\theta_k)\dot{\theta}_a, -\cos(\phi+\theta_a+\theta_k)\dot{\theta}_a, -\sin(\phi+2\theta_a)\dot{\theta}_a, -\cos(\phi+2\theta_a)\dot{\theta}_a, \ldots \\
&-\sin(\phi+2\theta_k)\dot{\theta}_a, -\cos(\phi+2\theta_k)\dot{\theta}_a, -\sin(\phi+2\theta_a+2\theta_k)\dot{\theta}_a, -\cos(\phi+2\theta_a+2\theta_k)\dot{\theta}_a, \ldots \\
&-\sin(\phi+2\theta_a+\theta_k)\dot{\theta}_a, -\cos(\phi+2\theta_a+\theta_k)\dot{\theta}_a, \ldots \\
&-\sin(\phi+\theta_a+2\theta_k)\dot{\theta}_a, -\cos(\phi+\theta_a+2\theta_k)\dot{\theta}_a]^T.
\end{aligned}
$$

2. WEIGHTING FACTORS FOR OPTIMIZATION PROBLEM

The optimization problem is re-stated below

$$
\begin{aligned}
\arg\min_{\alpha} \sum_j \{ & [\text{vGRF} \cdot U(q_j, p_j, \alpha) - Y_j]^T \cdot W_j(U, Y_j) \cdot [\text{vGRF} \cdot U(q_j, p_j, \alpha) - Y_j] \\
& + [U^{\text{B}}(q_j, p_j, \alpha) - Y_j^{\text{B}}]^T W_k [U^{\text{B}}(q_j, p_j, \alpha) - Y_j^{\text{B}}]\} \\
& + U(q_0, p_0, \alpha)^T W_r U(q_0, p_0, \alpha) + \Lambda ||W_s \alpha||_1.
\end{aligned}
$$

We set

$$
w_{\text{ankle}} = \begin{bmatrix} I(N_{\text{stance,early}}) & 0 & 0 & 0 \\ 0 & I(N_{\text{stance,middle}}) & 0 & 0 \\ 0 & 0 & 2I(N_{\text{stance,late}}) & 0 \\ 0 & 0 & 0 & 4I(N_{\text{swing}}) \end{bmatrix} \tag{S1}
$$

$$
w_{\text{knee}} = \begin{bmatrix} I(N_{\text{stance,early}}) & 0 & 0 & 0 \\ 0 & I(N_{\text{stance,middle}}) & 0 & 0 \\ 0 & 0 & 5I(N_{\text{stance,late}}) & 0 \\ 0 & 0 & 0 & 2I(N_{\text{swing}}) \end{bmatrix} \tag{S2}
$$

$$
W_j = \gamma_j \cdot \zeta \cdot \begin{bmatrix} w_{\text{ankle}} & 0 \\ 0 & w_{\text{knee}} \end{bmatrix}, \tag{S3}
$$

where $I(\cdot)$ represents the identity matrix, $N_{\text{stance,early}}$ and $N_{\text{stance,late}}$ represent the initial 15% and late 15% of stance phase boundaries. The coefficient γ_j changes according to the tasks. ζ depends on the exoskeleton and human inputs (U, Y_j) and equals to 10 if $\text{sign}(U(i) \cdot Y_j(i)) < 0$ for all sample index i, otherwise, $\zeta = 1$.

We also have

$$
w^k = \begin{bmatrix} I(N_{\text{stance,early}}) & 0 & 0 & 0 \\ 0 & 0 & 0 & 0 \\ 0 & 0 & I(N_{\text{stance,late}}) & 0 \\ 0 & 0 & 0 & I(N_{\text{swing}}) \end{bmatrix} \tag{S4}
$$

$$
W_k = \gamma_j \cdot \begin{bmatrix} w^k & 0 \\ 0 & w^k \end{bmatrix} \tag{S5}
$$

$$
W_r = 5I \tag{S6}
$$

$$
\Lambda = 0.5 \tag{S7}
$$

$$
W_s = \begin{bmatrix} I(N_{\text{Pot}}) & 0 \\ 0 & 10I(N_{\text{Gyro}}) \end{bmatrix}, \tag{S8}
$$

where N_{Pot} and N_{Gyro} represents the number of basis functions associated with the shaped potential energy and the shaped gyroscopic terms.

3. HUMAN SUBJECT RESULTS

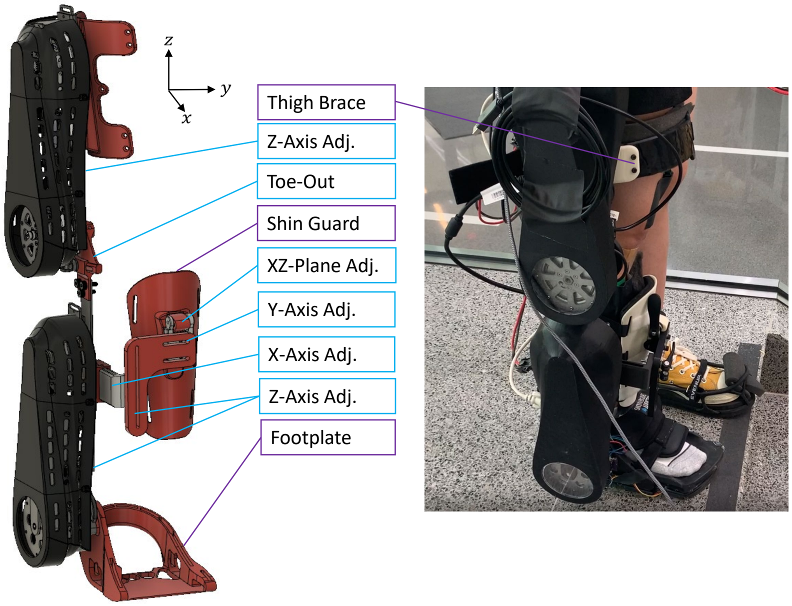

Fig. S1. CAD model of *Comex* knee-ankle exoskeleton. Four attachments and eight adjustments are capable of adjustment to secure the exoskeleton to the subject.

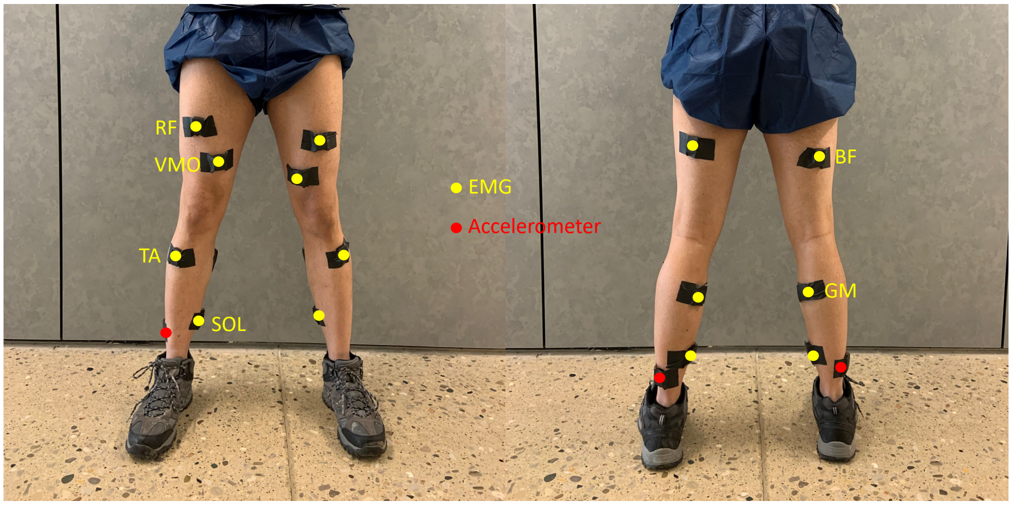

Fig. S2. Pictures with EMG measurement setup. The skin was prepared by removing the top layer of dead cells to reduce skin to electrode impedance. EMGs were placed and secured with tapes.

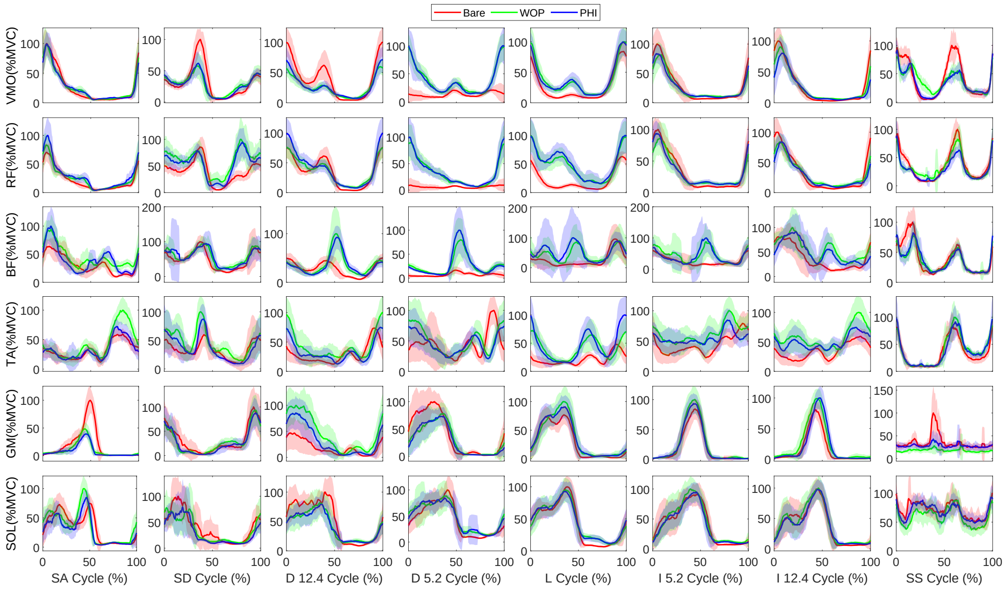

Fig. S3. Subject 2 EMG comparisons between bare and active modes (PHI and WOP methods) for each muscle (VMO, RF, BF, TA, GM and SOL) and task {Stairs Ascent/Descent (7in step height), Decline (−5.2°, −12.4°) at 0.6 m/s, level ground (1 m/s), Incline (5.2°, 12.4°) at 0.6 m/s, and Sit-Stand cycle (45 BPM)}. The red solid (bare), blue solid (PHI method), and green solid (WOP method) lines represent the time-normalized ensemble averages across all repetitions.

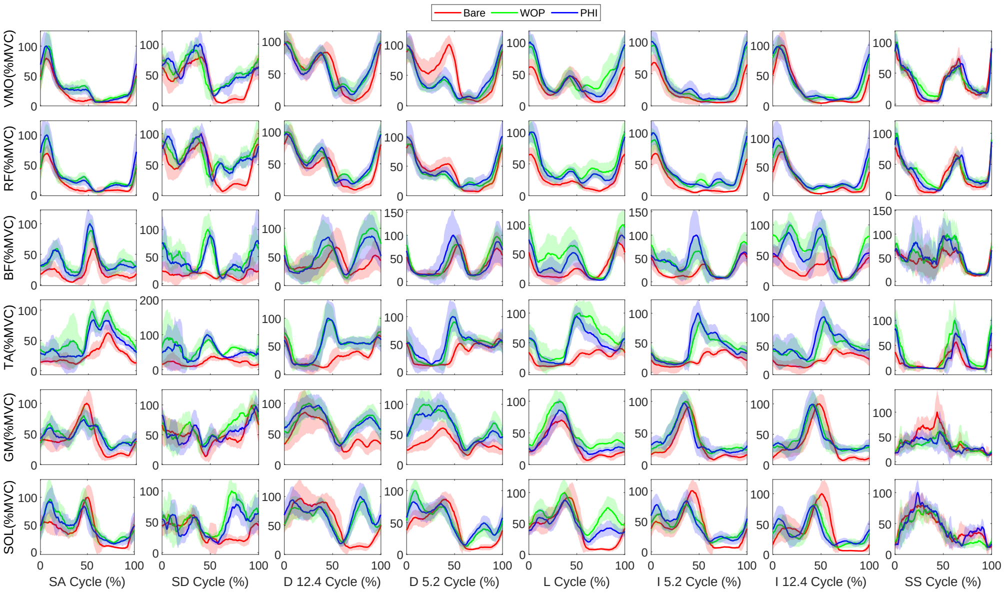

Fig. S4. Subject 3 EMG comparisons between bare and active modes (PHI and WOP methods) for each muscle (VMO, RF, BF, TA, GM and SOL) and task {Stairs Ascent/Descent (7in step height), Decline (−5.2°, −12.4°) at 0.6 m/s, level ground (0.8 m/s), Incline (5.2°, 12.4°) at 0.6 m/s, and Sit-Stand cycle (45 BPM)}. The red solid (bare), blue solid (PHI method), and green solid (WOP method) lines represent the time-normalized ensemble averages across all repetitions.

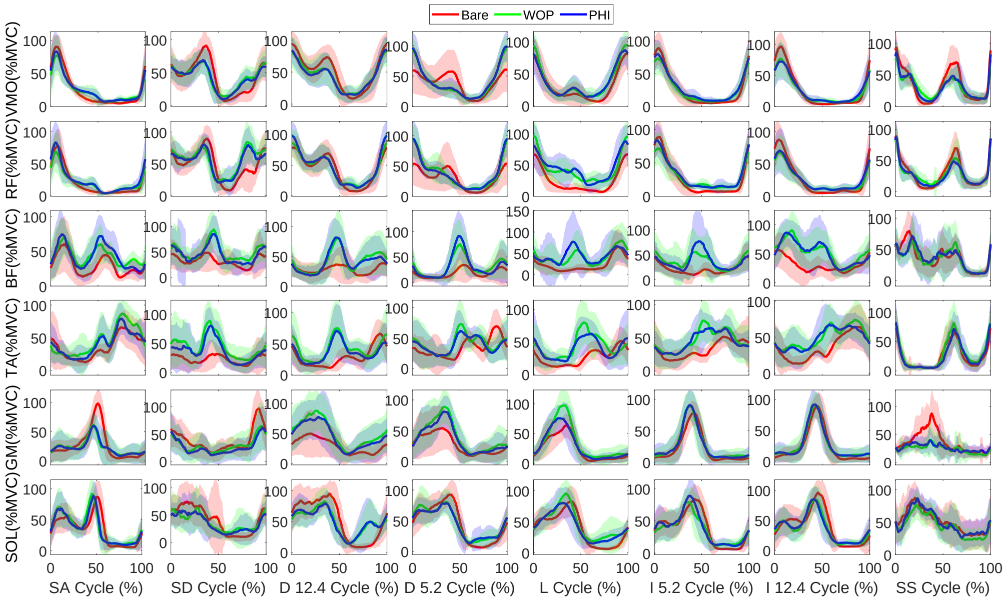

Fig. S5. Across-subjects averaged EMG comparisons between bare and active modes (PHI and WOP methods) for each muscle (VMO, RF, BF, TA, GM and SOL) and task {Stairs Ascent/Descent (7in step height), Decline ($-5.2°$, $-12.4°$) at 0.6 m/s, level ground (combined 0.8 m/s and 1 m/s), Incline ($5.2°$, $12.4°$) at 0.6 m/s, and Sit-Stand cycle (45 BPM)}. The red solid (bare), blue solid (PHI method), and green solid (WOP method) lines represent the time-normalized ensemble averages across all repetitions.

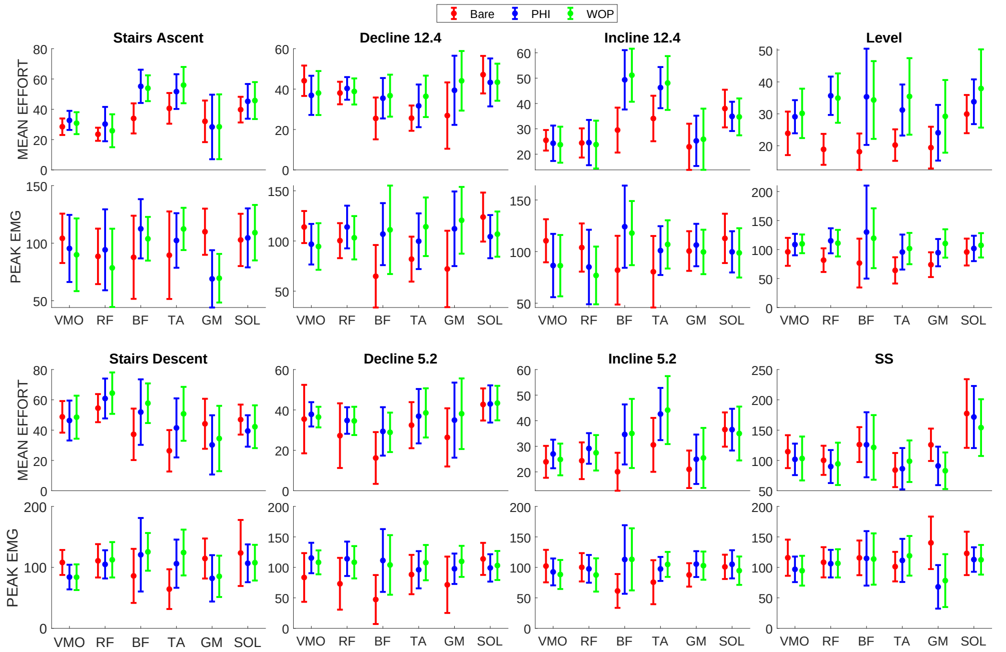

Fig. S6. Across-subjects mean effort (%MVC.S) and peak EMG (%MVC) comparisons for VMO, RF, BF, TA, GM and SOL: showing mean ($\pm$ SD) for different tasks and muscles (columns).

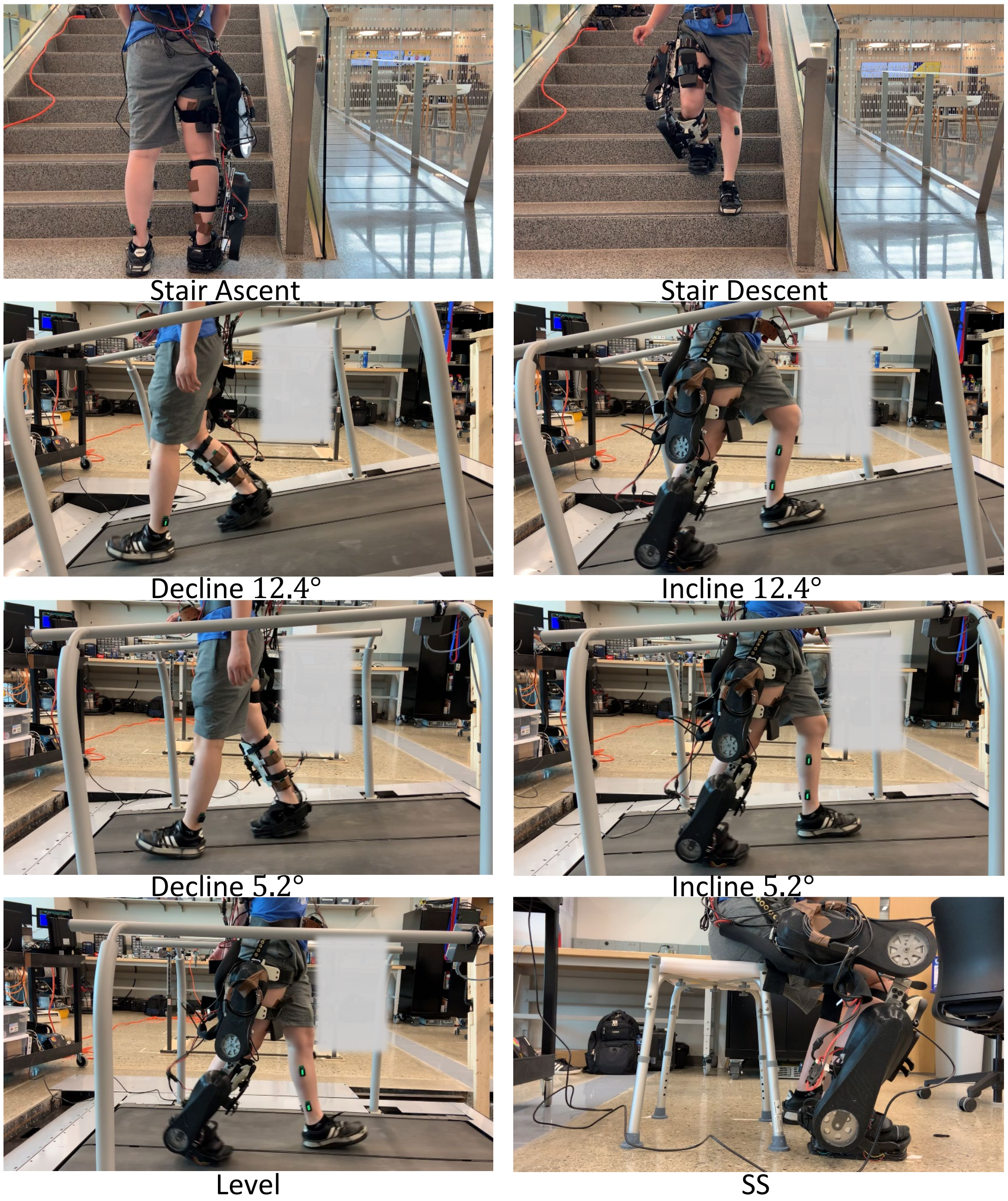

Fig. S7. The subject wore *Comex* knee-ankle exoskeleton and conducted tests for different tasks {Stairs Ascent/Descent (7in step height), Decline ($-5.2°$, $-12.4°$) at 0.6 m/s, Incline ($5.2°$, $12.4°$) at 0.6 m/s, level ground (1 m/s), and Sit-Stand cycle (45 BPM)}.

MEDIA

This supplementary material includes 1 video clip. The accompanying video demonstrates the versatility of the controller in providing biomimetic assistance across multiple activities.
